# Efficacy and safety of higher versus lower protein provision with meaningful dose separation in critically ill adults: a systematic review and meta-analysis of randomized controlled trials

**DOI:** 10.3389/fnut.2026.1893988

**Published:** 2026-07-31

**Authors:** Yun Zhou, Xiuxiu Hao, Run Pan, Tiantian Li, Zhiliang Yang, Zheng Wang, Yi Xiao

**Affiliations:** 1 Department of Cardiac Surgery, The People's Hospital of Kai Zhou District, Chongqing, China; 2 Department of Ultrasound Diagnosis, The Fifth Medical Center of Chinese PLA General Hospital, Beijing, China; 3 Orthopaedic Department, The People's Hospital of Kai Zhou District, Chongqing, China

**Keywords:** critical illness, intensive care unit, meta-analysis, mortality, nutrition support, protein provision, randomized controlled trial

## Abstract

**Background:**

Optimal protein provision in critically ill patients remains uncertain. We conducted a systematic review and meta-analysis of randomized controlled trials to evaluate the efficacy and safety of higher versus lower or standard protein provision in critically ill adults.

**Methods:**

Randomized controlled trials comparing higher versus lower/standard protein provision in critically ill adults were included. The primary analysis focused on trials with a between-group protein difference of at least 0.3 g/kg/day. Mortality was analyzed separately by time point, including 28/30-day, 60-day, 90-day, in-hospital, and ICU mortality. Secondary outcomes included mechanical ventilation duration, ICU and hospital length of stay, nutrition/metabolic markers, muscle-related outcomes, and safety. Risk ratios (RRs) and mean differences (MDs) were pooled using random-effects models. Certainty of evidence was assessed using GRADE.

**Results:**

Twenty-six studies were included. Higher protein provision did not significantly reduce mortality at any time point. For 28/30-day mortality, no statistically significant difference was observed, although the point estimate was lower with higher protein provision (RR = 0.82, 95% CI 0.58–1.17; *I*^2^ = 0%). No significant differences were observed for 60-day mortality (RR = 0.85, 95% CI 0.52–1.39; *I*^2^ = 51.1%), 90-day mortality (RR = 1.03, 95% CI 0.93–1.14; *I*^2^ = 0%), in-hospital mortality (RR = 1.04, 95% CI 0.78–1.39; *I*^2^ = 0%), or ICU mortality (RR = 0.84, 95% CI 0.57–1.26; *I*^2^ = 0%). Higher protein provision did not significantly shorten mechanical ventilation duration (MD = −0.74 days, 95% CI−1.92–0.43; *I*^2^ = 79.0%), ICU stay, or hospital stay. Higher protein provision was associated with a more favorable Day 3 nitrogen balance (MD = 6.39 g/day, 95% CI 2.77–10.01; *I*^2^ = 51.7%), but was not associated with a significant increase in gastrointestinal intolerance.

**Conclusions:**

Higher protein provision did not significantly reduce mortality or consistently improve clinical recovery outcomes in critically ill adults. It was associated with more favorable early nitrogen balance, but evidence for patient-centered benefit and safety remains limited.

## Introduction

Critical illness is characterized by profound metabolic stress, systemic inflammation, accelerated proteolysis, and rapid loss of lean body mass (1–4). During intensive care unit (ICU) admission, skeletal muscle wasting may occur early and progress rapidly, contributing to ICU-acquired weakness, delayed liberation from mechanical ventilation, prolonged ICU and hospital stay, impaired functional recovery, and poor long-term quality of life (4–6). Nutritional support is therefore considered an essential component of critical care management, aiming not only to provide energy substrates but also to attenuate protein catabolism and support tissue repair, immune function, and recovery (7).

Among macronutrients, protein has received increasing attention because critically ill patients often experience marked negative nitrogen balance and increased amino acid requirements. Current international guidelines generally recommend protein provision above that required for healthy adults, with commonly cited targets for critically ill patients ranging from approximately 1.2–2.0 g/kg/day; however, these targets vary across guidelines and may require adjustment in selected populations, including patients with trauma, burns, or obesity (7, 8). Nevertheless, these recommendations are largely based on physiological rationale, observational data, nitrogen balance studies, and expert consensus rather than consistent high-certainty randomized evidence. In clinical practice, achieving prescribed protein targets is also challenging because of feeding interruptions, gastrointestinal intolerance, hemodynamic instability, renal dysfunction, and differences between prescribed and actually delivered nutrition.

Randomized trials evaluating higher protein delivery in critically ill patients have produced inconsistent findings. As a result, the net clinical benefit and safety of higher protein provision in critically ill patients remain uncertain.

Previous reviews and meta-analyses have attempted to synthesize the evidence on higher versus lower protein nutrition in critical illness, but important methodological issues remain. First, trials differ substantially in both the absolute protein dose achieved in each treatment arm and the magnitude of dose separation between groups. Therefore, comparisons conducted entirely within a relatively high-protein range, such as 2.0 versus 1.5 g/kg/day, may address a different clinical question from comparisons between guideline-level and clearly lower protein provision, such as 1.2 versus 0.8 g/kg/day, even though both may be labeled as “higher versus lower protein” (9, 10). Second, several trials achieved only modest between-group separation in actually delivered protein, which may attenuate the contrast between higher and lower protein strategies and reduce the ability to detect a clinically meaningful treatment effect (9, 10). Third, mortality endpoints are reported at heterogeneous time points across critical care trials, including 28-/30-day, 60-day, 90-day, ICU, and in-hospital mortality. Because mortality endpoints reflect different follow-up windows and clinical contexts, we analyzed mortality according to prespecified time points to improve clinical interpretability, in accordance with guidance on defining and grouping outcomes for evidence synthesis (11).

To address these limitations, we conducted a systematic review and meta-analysis of randomized controlled trials comparing higher versus lower or standard protein nutrition support in critically ill adults. The main analysis focused on studies with a between-group protein difference of at least 0.3 g/kg/day, in order to ensure a clinically meaningful separation in protein exposure. Mortality outcomes were analyzed separately according to time point and definition. We also evaluated key secondary outcomes, including duration of mechanical ventilation, ICU and hospital length of stay, muscle mass and muscle function, nutrition and metabolic markers, and safety outcomes. In addition, we explored whether the effect of higher protein provision differed according to whether both groups achieved protein intake above 1.2 g/kg/day, both groups remained below this threshold, or the comparison crossed the 1.2 g/kg/day threshold. This approach aimed to clarify not only whether higher protein provision improves outcomes, but also whether the absolute level of protein intake modifies its potential benefit or harm in critically ill patients.

## Methods

### Protocol and registration

This systematic review and meta-analysis was conducted and reported in accordance with the PRISMA 2020 statement. The review included randomized controlled trials evaluating higher versus lower or standard protein provision in critically ill adult patients, with the primary analytic contrast defined as a between-group protein intake difference of at least 0.3 g/kg/day. For trials reporting amino acid dose rather than protein dose, amino acid provision was treated as protein-equivalent exposure only when the intervention contrast was clearly quantifiable, and this issue was considered when interpreting the findings. The protocol was prospectively registered in PROSPERO under the title “Effects of High Protein Feeding on Mortality, Muscle Mass, and Functional Outcomes in Critically Ill Patients: A Systematic Review and Meta-Analysis” (registration number: CRD420261363841).

### Eligibility criteria

Randomized controlled trials were eligible if they enrolled critically ill adult patients treated in an intensive care unit or comparable critical-care setting and compared a higher-protein nutrition strategy with a lower- or standard-protein strategy. Studies were eligible for the main quantitative analysis when the between-group difference in achieved, prescribed, or otherwise extractable protein provision was at least 0.3 g/kg/day. When both achieved and target protein intakes were reported, achieved protein delivery was prioritized. Studies were excluded if they were not randomized trials, did not include critically ill patients, did not compare different protein provision strategies, had a protein difference below 0.3 g/kg/day, did not provide quantifiable protein exposure in the comparator group, or lacked extractable outcome data. Studies without body-weight-normalized protein data were not included in the final study-characteristics table or threshold-based subgroup analyses. Studies were also excluded if they used multicomponent interventions or unequal co-interventions, such as combined protein supplementation with exercise or rehabilitation, that precluded isolation of the independent effect of protein provision. Trials in which between-group protein separation was limited to a short early phase and both groups subsequently received the same protein target were excluded because they were considered early versus delayed high-protein strategies rather than sustained higher-versus-lower protein comparisons.

### Search strategy

We systematically searched PubMed, Ovid Embase, the Cochrane Library, and China National Knowledge Infrastructure (CNKI) from database inception to May 1, 2026, without restrictions on language or publication date. The search strategy combined controlled vocabulary terms and free-text terms related to critical illness, intensive care, protein provision, clinical outcomes, and randomized controlled trials. The main search concepts included “ICU,” “intensive care,” “critical care,” “critically ill,” “critical illness,” “high protein,” “higher protein,” “protein supplementation,” “protein intake,” “protein provision,” “protein delivery,” “protein dose,” “amino acids,” “nitrogen balance,” “mortality,” “survival,” “length of stay,” “mechanical ventilation,” “muscle mass,” “randomized,” “randomized,” and “controlled trial”. For CNKI, equivalent Chinese search terms were used, including terms related to intensive care, critical illness, high protein intake, protein supplementation, enteral nutrition, parenteral nutrition, mortality, prognosis, and randomized controlled trials. Database-specific syntax was applied where appropriate. In addition, reference lists of relevant reviews and eligible studies were manually screened to identify additional records. The complete search strategies for each database are provided in Supplementary Table S1.

### Study selection

After duplicate removal, titles and abstracts were screened manually using Microsoft Excel by two reviewers independently. Full texts were retrieved for potentially eligible reports and assessed against the inclusion and exclusion criteria. Reasons for full-text exclusion were documented. Two reviewers independently screened titles, abstracts, and full texts, extracted data, and assessed risk of bias. Disagreements were resolved by discussion or consultation with a third reviewer.

### Data extraction

Data were extracted using a standardized extraction framework. Extracted information included study characteristics, country or region, study design, patient population, sample size, intervention and comparator nutrition strategies, target and achieved protein intakes, energy delivery when available, follow-up duration, and outcome data. When actual protein intake was unavailable, prescribed or protocol-specified protein targets were used and flagged accordingly. For continuous outcomes reported as medians and interquartile ranges, means and standard deviations were estimated using established approximation methods when appropriate (12). We also extracted ICU admission etiology or case mix from each included trial, including whether patients were enrolled from medical, surgical, trauma, neurological, respiratory, septic, or mixed ICU populations. When available, the number or proportion of surgical, trauma, and medical ICU patients was recorded. When etiology-specific counts were not reported, studies were classified according to the stated inclusion criteria or dominant study population.

We also extracted the mean or median age of participants in each treatment group when available. Age-based subgroup analysis was considered, particularly for older or potentially sarcopenic populations. However, formal subgroup analysis by age was planned only when age-stratified outcome data or at least two studies in each clearly age-defined subgroup were available.

### Risk-of-bias assessment

The risk of bias of included randomized trials was assessed using standard Cochrane risk-of-bias domains, including random sequence generation, allocation concealment, blinding of participants and personnel, blinding of outcome assessment, incomplete outcome data, selective reporting, and other potential sources of bias. Each domain was judged as low, unclear, or high risk of bias. Risk-of-bias findings were considered when interpreting outcome-specific certainty of evidence.

### Outcomes

The primary outcome was all-cause mortality. Mortality was analyzed separately according to time point and definition, including 28/30-day mortality, 60-day mortality, 90-day mortality, in-hospital mortality, and ICU mortality. These mortality outcomes were not pooled together because they represent different clinical time windows and outcome definitions.

Key secondary outcomes included duration of mechanical ventilation, ICU length of stay, hospital length of stay, muscle mass or muscle function outcomes, nutrition and metabolic outcomes, and safety outcomes. Muscle-related outcomes included peripheral muscle thickness or atrophy, diaphragm-related measures, and functional measures such as handgrip strength when available, and were pooled only when at least two eligible studies used sufficiently comparable measurements; otherwise, they were summarized descriptively. Nutrition and metabolic outcomes included nitrogen balance, albumin, blood urea nitrogen, and creatinine. Safety outcomes included gastrointestinal intolerance, gastric retention or high gastric residual volume, hyperglycemia, acute kidney injury, and kidney replacement therapy when extractable.

### Protein exposure classification and subgroup definitions

Because no consensus exists regarding the minimum clinically important between-group difference in protein delivery for ICU nutrition trials, we used a pragmatic threshold of 0.3 g/kg/day to define meaningful protein-dose separation. This threshold was not intended to represent a universally established biological cutoff. Rather, it was chosen for methodological reasons to reduce dilution of treatment effects from trials in which the nominal higher- and lower-protein groups achieved only small differences in actual protein delivery. In the context of commonly recommended protein targets for critically ill adults, such as approximately 1.2–2.0 g/kg/day, a difference of 0.3 g/kg/day represents a meaningful relative contrast and was considered sufficient to distinguish higher- versus lower-protein strategies for quantitative synthesis.

For threshold-based subgroup analyses, studies were classified according to whether the higher-protein and lower/standard-protein groups were above or below 1.2 g/kg/day. The double-high subgroup included comparisons in which both groups received at least 1.2 g/kg/day. The double-low subgroup included comparisons in which both groups received less than 1.2 g/kg/day. The cross-threshold subgroup included comparisons in which the higher-protein group received at least 1.2 g/kg/day and the comparator group received less than 1.2 g/kg/day. Actual delivered protein intake was used preferentially. If actual intake was unavailable, prescribed targets or converted estimates were used and identified as such.

### Energy-delivery classification

Because the effect of higher protein provision may depend on concurrent energy delivery, we additionally extracted the energy prescription, achieved energy intake when available, and the intended feeding paradigm from each trial. Trials were classified according to energy-delivery strategy as follows: energy-comparable or target-comparable, when energy delivery was intended to be similar between groups or no material between-group energy separation was reported; energy-non-comparable or caloric co-intervention, when higher protein provision was accompanied by clearly different energy delivery, including hypocaloric–hyperproteic strategies or higher-protein plus higher-energy interventions; and unclear/add-on, when total energy exposure could not be clearly categorized, such as trials using add-on intravenous amino acid supplementation on top of standard nutrition. This classification was used for exploratory subgroup or sensitivity analyses when data allowed.

### Subgroup analyses

Threshold-based subgroup analyses were performed according to a 1.2 g/kg/day protein-intake classification. The 1.2 g/kg/day threshold was selected because it approximates the lower boundary of commonly recommended protein targets for critically ill adults in contemporary nutrition guidelines. This threshold was used as a guideline-informed pragmatic cutoff for exploratory subgroup analysis and was not intended to represent a biologically validated cutoff. Studies were classified as double-high, double-low, or cross-threshold comparisons according to whether protein intake in the higher-protein and comparator groups was ≥1.2 g/kg/day or < 1.2 g/kg/day. Actual delivered protein intake was prioritized; when actual intake was unavailable, prescribed targets or extractable estimates were used. Formal threshold-based subgroup comparisons were performed only when at least two subgroups were available and each subgroup contained at least two studies.

In addition, exploratory subgroup analyses according to energy-delivery strategy were considered for outcomes with sufficient data. To avoid overinterpretation of sparse data, formal subgroup comparisons were performed only when at least two subgroups were available and each subgroup contained at least two studies. When an energy-defined subgroup contained only one study, no formal subgroup comparison was performed; instead, the finding was presented descriptively or in the Supplementary Materials when appropriate. Such findings were not used to draw definitive conclusions regarding subgroup effects.

Etiology-based subgroup analyses were considered according to ICU admission category or dominant case mix, including trauma/surgical, medical, neurological, respiratory/sepsis, and mixed ICU populations. Formal etiology-based subgroup comparisons were planned only when at least two studies were available in at least two etiology-defined subgroups for a given outcome. When studies enrolled mixed ICU populations without etiology-specific outcome data, they were summarized descriptively rather than used to draw subgroup-specific conclusions.

### Sensitivity analyses

Sensitivity analyses were performed to assess the robustness of pooled estimates and to explore potential sources of heterogeneity. These included leave-one-out analyses for outcomes with a sufficient number of studies, assessment of the influence of studies requiring median/interquartile range conversion, and evaluation of whether individual trials materially changed the pooled estimates. When formal subgroup analysis by energy-delivery strategy was not feasible because one or more subgroups contained too few studies, sensitivity analyses were conducted where appropriate by excluding trials with clearly non-comparable energy delivery or unclear/add-on energy exposure. For outcomes in which the energy-comparable subgroup was already presented within an exploratory subgroup analysis, the subgroup subtotal was considered to also represent the sensitivity analysis excluding energy-non-comparable trials, and no duplicate sensitivity forest plot was generated.

### Statistical analysis

Risk ratios (RRs) with 95% confidence intervals (CIs) were used for dichotomous outcomes. Mean differences (MDs) with 95% CIs were used for continuous outcomes measured on the same scale. Continuous outcomes were pooled using mean differences when measured on comparable scales. Outcomes with substantial differences in measurement site, method, or time point were not quantitatively pooled and were summarized descriptively. For duration outcomes, MDs were calculated as the higher-protein group minus the lower/standard-protein group; therefore, negative values indicate shorter duration or length of stay in the higher-protein group. For nitrogen balance and albumin, positive MDs indicate higher values in the higher-protein group.

Random-effects models were used for meta-analysis because clinical and methodological heterogeneity across trials was anticipated. Heterogeneity was assessed using the *I*^2^ statistic and the Cochran Q statistic. *I*^2^ values were interpreted in the context of the number of studies, effect directions, and clinical diversity. Outcomes with fewer than two studies were not pooled. Single-study outcomes were summarized descriptively. Meta-analyses were performed using Review Manager 5.4.

### Publication bias

Publication bias was planned to be assessed using funnel plots when at least 10 studies were available for an outcome. For outcomes with fewer than 10 studies, publication bias was not formally assessed because funnel plot asymmetry is unreliable with small numbers of studies.

### Certainty of evidence

The certainty of evidence for key outcomes was assessed using the GRADE approach. Randomized trials were initially rated as high-certainty evidence and were downgraded according to risk of bias, inconsistency, indirectness, imprecision, and publication bias. Certainty was categorized as high, moderate, low, or very low. A Summary of Findings table is presented in the Table 1, and the complete GRADE Evidence Profile is provided in Supplementary Table S2.

**Table 1:** GRADE summary of findings for higher versus lower protein provision in critically ill adults.

Outcome	No. of participants (studies)	Effect estimate (95% CI)	Certainty of evidence (GRADE)	Comments
28/30–day all–cause mortality	488 (6 RCTs)	RR 0.82 (95% CI 0.58–1.17)	⊕⊕⊕◯ Moderate	Downgraded one level for imprecision because the 95% CI crossed the line of no effect.
60–day all–cause mortality	1,406 (3 RCTs)	RR 0.85 (95% CI 0.52–1.39)	⊕⊕◯◯ Low	Downgraded for moderate heterogeneity and a wide confidence interval crossing the line of no effect.
90–day all–cause mortality	4,121 (5 RCTs)	RR 1.03 (95% CI 0.93–1.14)	⊕⊕⊕◯ Moderate	Downgraded one level for some concerns in risk of bias across included trials; the estimate was precise and heterogeneity was low.
In–hospital mortality	620 (2 RCTs)	RR 1.04 (95% CI 0.78–1.39)	⊕⊕◯◯ Low	Downgraded for risk–of–bias concerns and imprecision due to few studies and confidence interval crossing no effect.
ICU mortality	161 (2 RCTs)	RR 0.84 (95% CI 0.57–1.26)	⊕◯◯◯ Very low	Downgraded for risk–of–bias concerns and very serious imprecision due to a small sample size and wide confidence interval.
Duration of mechanical ventilation	3,973 (8 RCTs)	MD−0.74 days (95% CI−1.92–0.43)	⊕◯◯◯ Very low	Downgraded for risk–of–bias concerns, high heterogeneity, and imprecision because the confidence interval crossed no effect.
ICU length of stay	1,163 (9 RCTs)	MD−0.17 days (95% CI−1.39–1.04)	⊕⊕◯◯ Low	Downgraded for risk–of–bias concerns and imprecision because the confidence interval crossed no effect.
Hospital length of stay	820 (5 RCTs)	MD 1.13 days (95% CI−3.94–6.20)	⊕◯◯◯ Very low	Downgraded for risk–of–bias concerns, heterogeneity, and very serious imprecision due to a wide confidence interval.
Day 3 nitrogen balance	140 (2 RCTs)	MD 6.39 g/day (95% CI 2.77–10.01)	⊕◯◯◯ Very low	Downgraded for risk–of–bias concerns, moderate heterogeneity, indirectness because nitrogen balance is a surrogate/mechanistic outcome, and few studies.
Gastrointestinal intolerance composite	990 (2 RCTs)	RR 1.00 (95% CI 0.32–3.16)	⊕◯◯◯ Very low	Downgraded for risk–of–bias concerns, heterogeneity, varying definitions of gastrointestinal intolerance, and very wide confidence interval.

CI, confidence interval; GRADE, grading of recommendations assessment, development and evaluation; ICU, intensive care unit; MD, mean difference; RCT, randomized controlled trial; RR, risk ratio. RR < 1 favors higher protein provision for dichotomous outcomes; negative MD favors higher protein provision for duration outcomes; positive MD indicates higher Day 3 nitrogen balance in the higher–protein group. GRADE certainty levels are shown as ⊕⊕⊕◯, moderate; ⊕⊕◯◯, low; and ⊕◯◯◯, very low.

## Results

### Study selection

**Figure 1:**
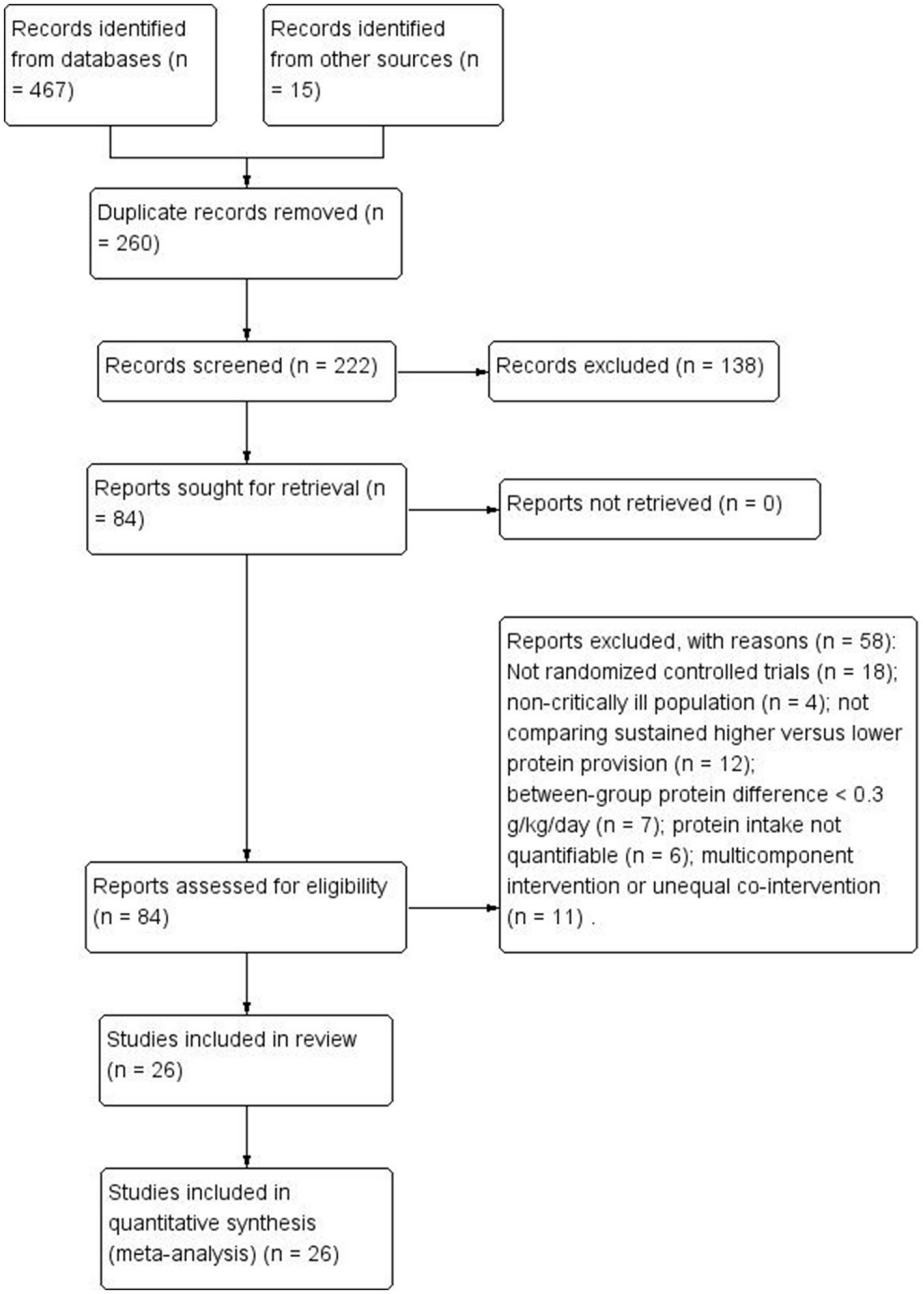
PRISMA flow diagram of literature search and study selection.

A total of 482 records were identified, including 467 records from database searches and 15 records from other sources. After removal of 260 duplicates, 222 records were screened by title and abstract. Of these, 138 records were excluded, and 84 reports were sought for full-text retrieval. No reports were unavailable for retrieval. Therefore, 84 full-text reports were assessed for eligibility. Fifty-eight reports were excluded for the following reasons: not randomized controlled trials (*n* = 18), non-critically ill population (*n* = 4), not comparing sustained higher versus lower protein provision (*n* = 12), between-group protein difference < 0.3 g/kg/day (*n* = 7), protein intake not quantifiable (*n* = 6), and multicomponent intervention or unequal co-intervention (*n* = 11). Finally, 26 studies were included in the systematic review and quantitative synthesis (Figure 1).

### Study characteristics

The included randomized controlled trials enrolled critically ill adult patients from mixed medical-surgical ICUs, mechanically ventilated populations, trauma or traumatic brain injury cohorts, severe pneumonia patients, severe stroke patients, and patients requiring prolonged mechanical ventilation. The nutrition interventions varied across studies and included high-protein enteral formulas, enteral protein supplementation, parenteral amino acid supplementation, and protocolized nutrition strategies targeting higher protein provision.

The between-group protein separation was at least 0.3 g/kg/day in the studies included in the main quantitative analyses. Actual delivered protein intake was used preferentially for exposure classification. When actual intake was not available, protocol-specified or prescribed targets were used. According to the 1.2 g/kg/day threshold, most studies were classified as cross-threshold comparisons, in which the higher-protein group received ≥1.2 g/kg/day and the comparator group received < 1.2 g/kg/day. A smaller number of studies were classified as double-high comparisons, in which both groups received ≥1.2 g/kg/day. Only one study was classified as double-low. The core characteristics of the 26 studies contributing to the quantitative synthesis are summarized in Table 2 (13–38), and detailed etiology-specific patient numbers and energy-delivery strategies are provided in Supplementary Table S3.

**Table 2:** Baseline characteristics and protein interventions of randomized controlled trials included in the quantitative synthesis.

References (Region)	ICU Admission etiology/Case mix	N (Higher/Comparator)	Age, years (Higher vs. Comparator)	Protein dose (Higher vs. Comparator)	Energy delivery (Higher vs. Comparator)	Feeding paradigm	Primary outcome/Key endpoint
Arabi et al. (13); Saudi Arabia	Mixed ICU; predominantly medical and non–operative trauma	21/19	43.6 (24.6, 63.6) vs. 58.9 (25.7, 73.5)	1.30 vs. 0.77 g/kg/day | Cross–threshold	Target 40–60% estimated energy expenditure in both groups	Energy–comparable permissive underfeeding high–protein strategy	Feasibility and protein delivery
Azevedo et al. (14); Brazil	Mixed medical–surgical ICU	57/63	65.0 (18.8) vs. 67.4 (18.9)	1.69 vs. 1.13 g/kg/day | Cross–threshold	IC–guided energy in high–protein arm vs. 25 kcal/kg/day target in comparator; received calories not significantly different	Energy–comparable high–protein strategy; no significant difference in calories received	Physical quality of life/clinical recovery
Badpeyma et al. (15); Iran	Mixed ICU; poisoning, respiratory, neurologic, sepsis and other admissions	28/28	54.32 ± 19.25 vs. 58.18 ± 18.56	1.67 vs. 0.93 g/kg/day | Cross–threshold	Target 25 kcal/kg/day in both groups	Energy–comparable high–protein strategy	60–day mortality and clinical outcomes
Bels et al. (16); Belgium; Netherlands	Mixed ventilated ICU; medical, surgical, respiratory, cardiovascular, neurologic and trauma diagnoses	470/465	62 (14) vs. 63 (14)	Target 2.0 vs. 1.3 g/kg/day; achieved approx. 1.87 vs. 1.19 g/kg/day | Target–based double–high; achieved–dose borderline	Isocaloric EN formulas, 1.3 kcal/ml in both groups; delivered energy similar	Isocaloric high–protein formula strategy	EQ−5D−5L health utility over 180 days
Chapple et al. (17); Australia; New Zealand	Mixed critically ill patients receiving enteral nutrition; etiology–specific counts not separately reported	58/58	60 [50, 72] vs. 61 [46, 68]	Approx. 1.50 vs. 1.00 g/kg/day | Cross–threshold	Energy target according to usual/site practice in both groups; achieved energy incompletely reported	Energy–comparable high–protein formula strategy; detailed achieved energy incompletely reported	Feasibility and protein delivery
Liu et al. (18); China	Severe pneumonia; mechanically ventilated medical ICU population	60/60	Overall 61.4 ± 12.01	1.80 vs. 1.20 g/kg/day | Double–high	Target 25 kcal/kg/day based on ideal body weight in both groups	Isocaloric high–protein strategy	Muscle/diaphragm ultrasound outcomes
Hepduman et al. (19); Turkiye	Mechanically ventilated ICU; predominantly pneumonia/respiratory admissions	21/21	73.62 ± 11.41 vs. 64.38 ± 18.16	1.50 vs. 1.10 g/kg/day | Cross–threshold	Target calories intended to be comparable; achieved energy incompletely reported	Energy–comparable high–protein strategy; achieved energy/protein incompletely reported	Diaphragm thickness
Doig et al. (20); Australia; New Zealand	Mixed ICU at risk of renal dysfunction; medical, surgical, emergency and trauma diagnoses	239/235	62.7 ± 16.6 vs. 63.3 ± 15.4	Approx. 2.00 vs. 0.75 g/kg/day | Cross–threshold	Standard nutrition in both groups; IV amino acids added energy in intervention arm	Protein/amino–acid supplementation added to standard care; total energy slightly increased by amino acids	Kidney function/renal outcomes
Dresen et al. (21); Germany	Long–term immobilized ICU; mixed surgical, ARDS and trauma–surgery diagnoses	21/21	66 ± 16 vs. 64 ± 15	1.50 vs. 1.00 g/kg/day | Cross–threshold	Target 100% measured resting energy expenditure by indirect calorimetry in both groups	Isocaloric high–protein strategy	Quadriceps muscle loss
Uyar et al. (22); Turkiye	Mechanically ventilated ICU; mainly respiratory and neurologic admissions	25/24	61.72 ± 21.95 vs. 59.79 ± 23.82	2.00 vs. 1.20 g/kg/day | Double–high	Caloric target/protocol not clearly extractable	Likely energy–comparable high–protein strategy; energy details incompletely reported	Diaphragm function and muscle thickness
Fetterplace et al. (23); Australia	Mixed ICU; neurologic, trauma, respiratory, sepsis and cardiac–surgery admissions	30/30	55 (13) vs. 57 (16)	1.20 vs. 0.75 g/kg/day | Cross–threshold	Target 25 kcal/kg/day in both groups; delivered energy 21 vs. 18 kcal/kg/day from nutrition and 23 vs. 21 kcal/kg/day total	High–protein plus higher–energy co–intervention	Protein delivery/feasibility and muscle loss
Garcia–de–Lorenzo et al. (24); Spain	Septic ICU patients receiving total parenteral nutrition	25/22	50.3 ± 4 vs. 49.5 ± 4.2	1.50 vs. 1.10 g amino acids/kg/day | Cross–threshold^*^	Isocaloric TPN: 24 non–protein kcal/kg/day in both groups	Isocaloric parenteral amino–acid strategy	Nitrogen balance and metabolic response
O'Keefe et al. (25); United States	Trauma and surgical ICU patients	244/256	45.1 (16.5) vs. 47.4 (17.4)	1.20 vs. 0.60 g/kg/day | Cross–threshold	Protein supplementation independent of enteral calories; detailed energy delivery incompletely reported	Protein supplementation added to usual EN; energy exposure incompletely reported	Transthyretin and clinical complications
Elkalawy et al. (26); Egypt; United States	Traumatic brain injury; mechanically ventilated neurotrauma ICU population	53/49	40 ± 6 vs. 39 ± 7	1.80 vs. 1.20 g/kg/day | Double–high	Energy individualized by indirect calorimetry in both groups; group–level achieved energy incompletely extractable	High–protein formula under IC–guided energy delivery; achieved energy details incompletely extractable	Muscle mass and ICU recovery outcomes
Heyland et al. (27); International	Mixed nutritionally high–risk ventilated ICU; respiratory, neurologic, sepsis, trauma and other diagnoses	642/648	57 [17 (18–95)] vs. 57 [17 (18–93)]	Approx. 1.60 vs. 0.90 g/kg/day | Cross–threshold	Energy delivery guided by standard ICU nutrition practice; no distinct hypocaloric protocol	Protein–dose strategy without a distinct hypocaloric protocol	Time–to–discharge–alive and 60–day mortality
Danielis et al. (28); Italy	Critically ill ICU patients; detailed etiology counts not separately reported	19/21	66.0 (57.0–72.0) vs. 63.0 (46.0–70.0)	1.80 vs. 0.80 g/kg/day | Cross–threshold	Nutrition set to meet energy requirements in both groups; no energy difference except Day 1	Energy–comparable high–protein strategy; no energy difference except Day 1	Nitrogen balance
Summers et al. (29); Australia; New Zealand	Mixed ICU receiving enteral nutrition; respiratory, neurologic, cardiovascular, trauma, gastrointestinal and sepsis diagnoses	1681/1716	61 (48–70) vs. 61 (48–71)	1.33 vs. 0.84 g/kg/day | Cross–threshold	Achieved energy 17 kcal/kg IBW/day and 13 kcal/kg actual BW/day in both groups	Isocaloric high–protein enteral strategy	Hospital–free days/90–day survival
Miri et al. (30); Iran	Mechanically ventilated ICU; mainly neurologic, trauma, unspecified and other diagnoses	50/50	51.5 ± 14.2 vs. 52.2 ± 10.8	1.50 vs. 1.00 g/kg/day | Cross–threshold	Target energy described as comparable; achieved energy incompletely reported	Energy–comparable high–protein strategy; achieved energy incompletely reported	Biceps muscle thickness and nitrogen balance
Nakamura et al. (31); Japan	Mixed critically ill patients; sepsis, stroke, post–surgery, respiratory failure and trauma reported as comorbid/basic diseases	60/57	68.3 ± 14.3 vs. 67.9 ± 14.9	1.50 vs. 0.80 g/kg/day | Cross–threshold	Target energy 20 kcal/kg/day in both groups	Isocaloric high–protein strategy	Femoral muscle volume
Rugeles et al. (32); Colombia	Critically ill adults receiving enteral nutrition; respiratory and CNS disorders predominant	40/40	53.3 (19.5) vs. 55.7 (19.5)	1.40 vs. 0.76 g/kg/day | Cross–threshold	Hypocaloric–hyperproteic vs. isocaloric prescription: 15 vs. 25 kcal/kg/day; achieved about 12 vs. 14 kcal/kg/day	Hypocaloric–hyperproteic strategy	SOFA score and clinical outcomes
Jakob et al. (33); Switzerland	Medical and surgical ICU long–stayers receiving tube feeding; detailed etiology counts not separately reported	46/44	65.3 (52.6–75.3) vs. 61.6 (48.6–71.3)	1.13 vs. 0.80 g/kg/day | Double–low	IC–guided caloric goal; achieved energy 18.0 vs. 19.7 kcal/kg/day	Isocaloric high–protein formula strategy	GI tolerance/diarrhea
van Zanten et al. (34); Netherlands; Belgium	Overweight mixed ICU; medical, surgical and trauma admissions	22/22	63.9 (13.3) vs. 60.8 (15.2)	1.49 vs. 0.76 g/kg/day | Cross–threshold	Isocaloric formulas, 1.25 kcal/ml in both groups; no increase in energy intake	Isocaloric high protein–to–energy formula strategy	Protein intake, GI tolerance, safety
Parasuraman et al. (35); India	Mechanically ventilated ICU; predominantly pneumonia	15/15	35.8 ± 13.73 vs. 36.6 ± 13.9	1.50 vs. 1.00 g/kg/day | Cross–threshold	Normocaloric EN target about 25 kcal/kg/day in both groups	Isocaloric/normocaloric high–protein strategy	Muscle ultrasound outcomes
Wu et al. (36); China	Mechanically ventilated surgical ICU patients	42/49	NR in article PDF	2.50–3.00 vs. approx. 1.20–1.50 g/kg/day | Double–high	Target calories 25 kcal/kg/day for 7 days in both groups	Isocaloric high–protein strategy	Survival, VAP, ICU–AW, safety
Zhang et al. (37); China	Prolonged mechanical ventilation; mixed medical/surgical admissions with frequent severe sepsis	20/21	64.45 ± 16.17 vs. 69.24 ± 18.15	1.70 vs. 1.06 g/kg/day | Cross–threshold	Actual energy 33.46 +/– 2.78 vs. 25.75 +/– 4.81 kcal/kg/day	High–protein plus higher–energy co–intervention	Diaphragm atrophy and respiratory mechanics
Zhou et al. (38); China	Severe stroke receiving isocaloric enteral nutrition	25/26	Overall 65.69 ± 15.63	1.56 vs. 1.20 g/kg/day | Double–high	Equal non–protein calories 104.5 kJ/kg/day in both groups	Isocaloric high–protein formula strategy	Albumin, prealbumin, hypoproteinemia, survival
Total	26 RCTs	4,014/4,060; total 8,074	—	—	—	—	—

Double–high indicates that both groups received ≥1.2 g/kg/day protein; double-low indicates that both groups received < 1.2 g/kg/day protein; cross-threshold indicates that the higher-protein group received ≥1.2 g/kg/day and the comparator group received < 1.2 g/kg/day. Energy delivery is reported as achieved intake when available and as prescribed or target intake when achieved intake was unavailable. ICU admission etiology/case mix was classified according to the enrolled population or dominant admission category reported by each trial. Abbreviations: EN, enteral nutrition; PN, parenteral nutrition; TPN, total parenteral nutrition; IV, intravenous; ICU, intensive care unit; IBW, ideal body weight; ABW, actual body weight; IC, indirect calorimetry; EMS, electrical muscle stimulation; GI, gastrointestinal; VAP, ventilator-associated pneumonia; ICU-AW, intensive care unit-acquired weakness.

* Garcia-de-Lorenzo 1997 reported amino acid dose rather than protein dose and used a three-arm design; classification was based on the final analytic contrast used in the meta-analysis.

The mean or median age of participants was extracted and added to Table 2 when available. Most trials enrolled middle-aged to older critically ill adults, but few studies specifically targeted older adults or sarcopenic patients, and age-stratified treatment effects were generally not reported. Therefore, formal subgroup analysis by age was not feasible.

### Risk of bias

**Figure 2:**
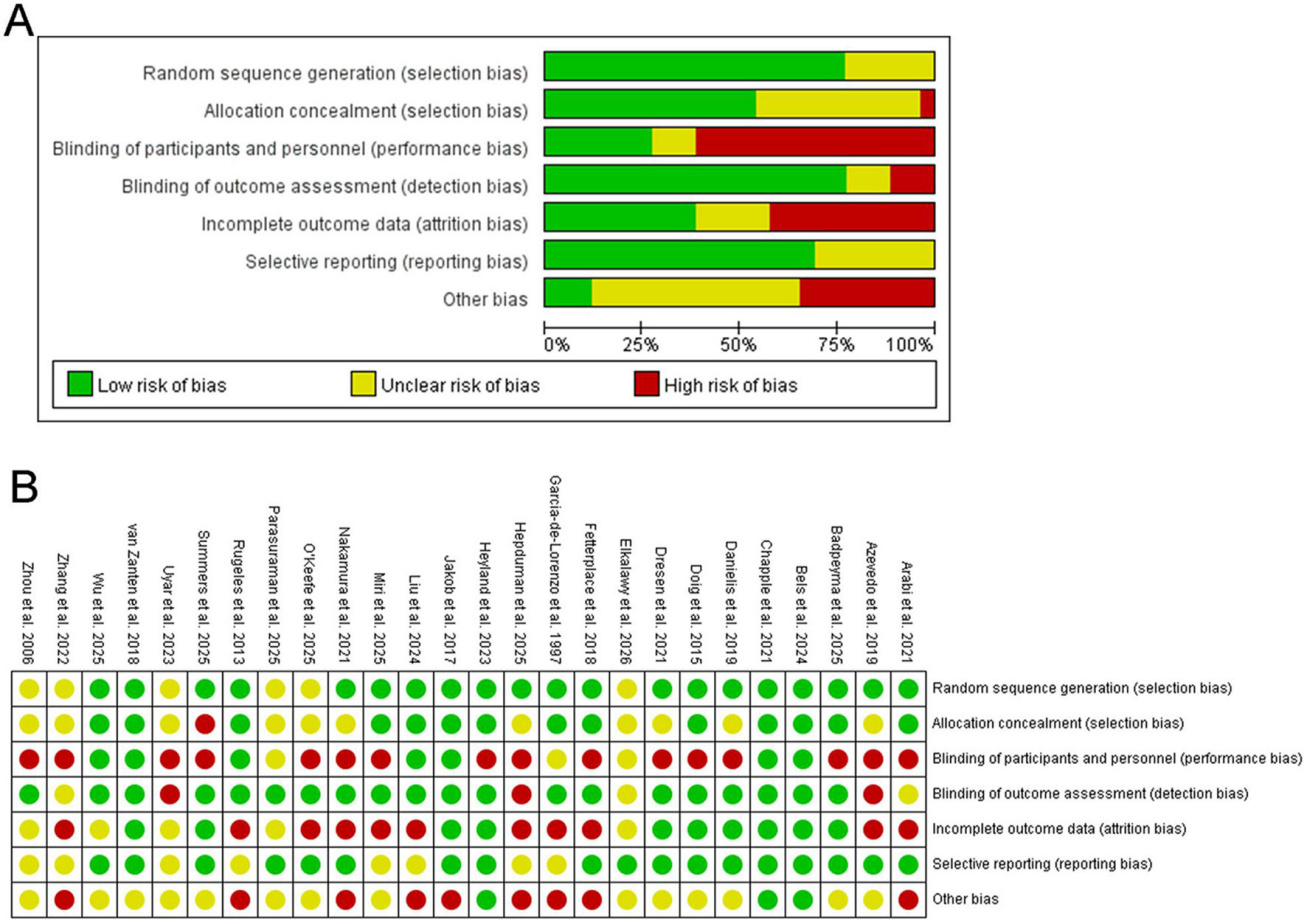
Cochrane risk-of-bias assessment of included randomized controlled trials. **(A)** overall risk-of-bias assessment showing the proportion of studies judged as low, unclear, or high risk of bias for each domain. **(B)** study-level risk-of-bias summary showing judgments for each trial across individual domains. Green indicates low risk of bias, yellow indicates unclear risk of bias, and red indicates high risk of bias.

The overall methodological quality of the included randomized trials was variable. Some trials reported adequate random sequence generation, allocation concealment, and blinded outcome assessment, whereas others had unclear or high risk of bias because of insufficient reporting, open-label intervention design, incomplete blinding, or selective/incomplete outcome reporting (Figures 2A, B). Mortality outcomes were generally considered less susceptible to detection bias because they were objective outcomes. In contrast, functional, nutritional, and muscle-related outcomes were more vulnerable to performance and detection bias because of the nature of nutrition interventions and the use of operator-dependent measurements such as ultrasound-based muscle assessments. Therefore, risk-of-bias concerns were considered when interpreting outcome-specific certainty of evidence.

### Primary outcome: mortality

Mortality outcomes were analyzed separately according to their prespecified time points or definitions. We did not combine 28/30-day, 60-day, 90-day, in-hospital, and ICU mortality in a single pooled analysis.

**Figure 3:**
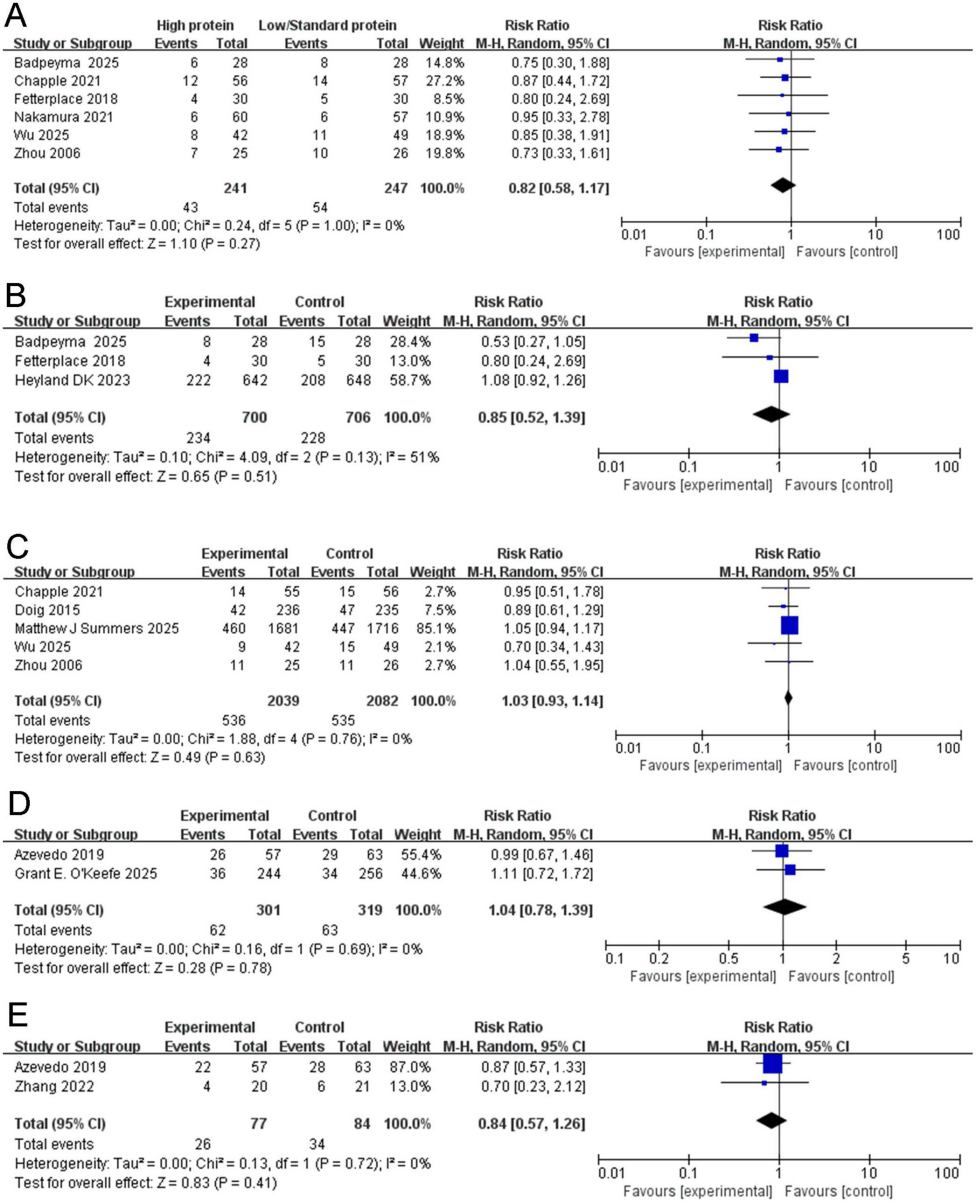
Forest plots of mortality outcomes. **(A)** 28/30-day all-cause mortality; **(B)** 60-day all-cause mortality; **(C)** 90-day all-cause mortality; **(D)** in-hospital mortality; **(E)** ICU mortality. Risk ratios compare higher-protein with lower/standard-protein provision; values less than 1 favor higher-protein provision.

Six RCTs reported 28/30-day all-cause mortality. Overall, higher protein provision did not significantly reduce mortality at any analyzed time point. For 28/30-day mortality, the point estimate was below 1.0, but the confidence interval crossed the line of no effect and the result remained statistically inconclusive (RR = 0.82, 95% CI 0.58–1.17; *I*^2^ = 0%; Figure 3A). Because only one study had clearly non-comparable energy delivery, formal subgroup analysis by energy strategy was not feasible. Excluding this study in a sensitivity analysis did not materially change the result (RR = 0.82, 95% CI 0.57–1.19; *I*^2^ = 0%, Supplementary Figure S1A).

Three RCTs reported 60-day all-cause mortality. No significant difference was observed between higher and lower/standard protein provision (RR = 0.85, 95% CI 0.52–1.39; *I*^2^ = 51.1%; Figure 3B). Because only one study had clearly non-comparable energy delivery, formal subgroup analysis was not performed; exclusion of this study also yielded a statistically nonsignificant result (RR = 0.82, 95% CI 0.42–1.61; *I*^2^ = 74%; Supplementary Figure S1B). Five RCTs reported 90-day all-cause mortality. Higher protein provision did not reduce 90-day mortality (RR = 1.03, 95% CI 0.93–1.14; *I*^2^ = 0%; Figure 3C). Formal energy-strategy subgroup analysis was not feasible because no study clearly used a hypocaloric–hyperproteic strategy and the energy-non-comparable subgroup contained too few studies. Exclusion of the add-on amino acid trial with unclear total energy effect did not materially change the result (RR = 1.04, 95% CI 0.93–1.15; *I*^2^ = 0%; Supplementary Figure S1C).

For in-hospital mortality, two RCTs were included, and no significant difference was found between groups (RR = 1.04, 95% CI 0.78–1.39; *I*^2^ = 0%; Figure 3D). ICU mortality was also not significantly different between groups based on two RCTs (RR = 0.84, 95% CI 0.57–1.26; *I*^2^ = 0%; Figure 3E).

### Clinical recovery outcomes

Clinical recovery outcomes included duration of mechanical ventilation, ICU length of stay, and hospital length of stay. Mean differences were calculated as the higher-protein group minus the lower/standard-protein group; therefore, negative values favored higher protein provision.

**Figure 4:**
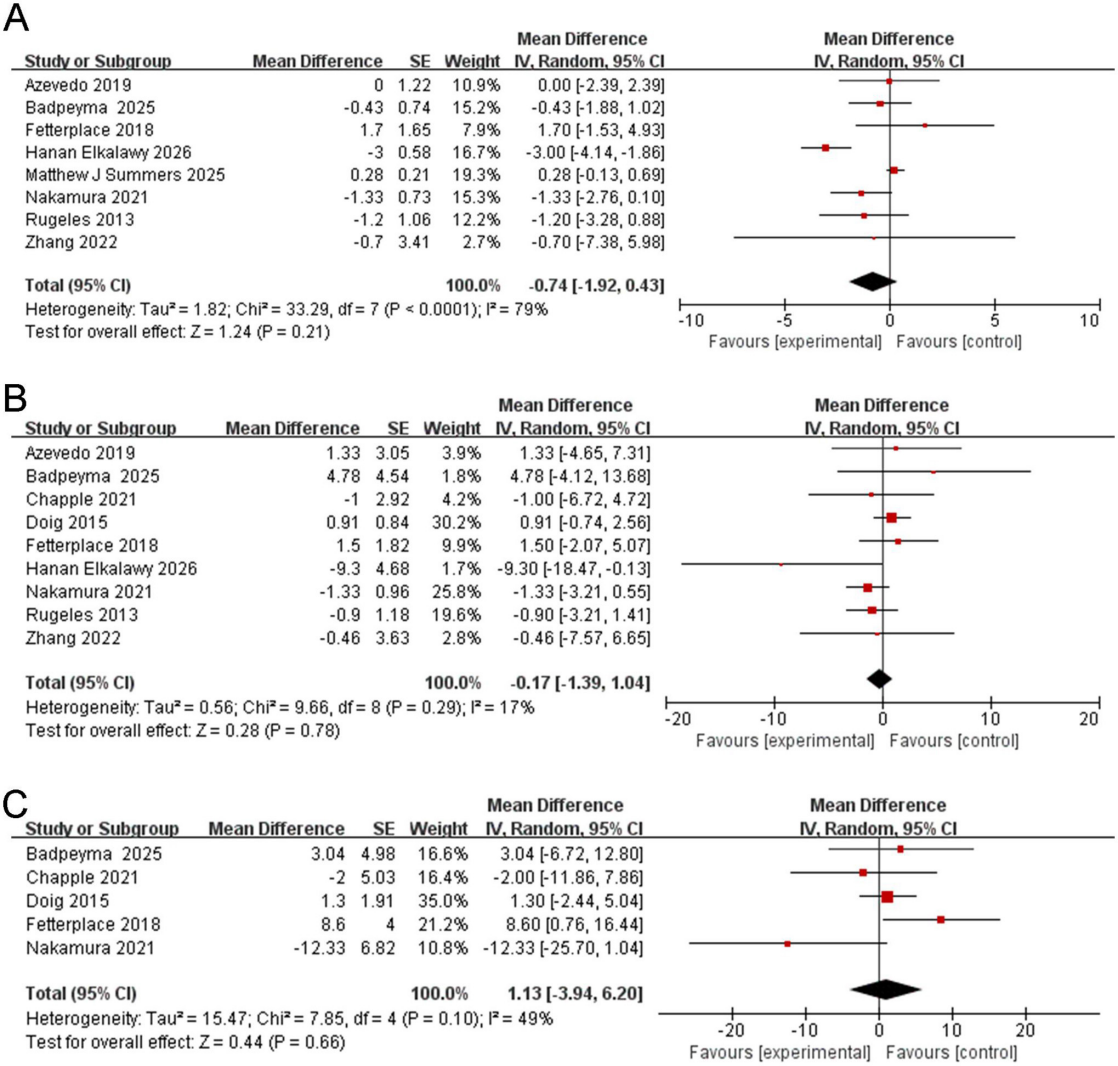
Forest plots of clinical recovery outcomes. **(A)** duration of mechanical ventilation; **(B)** ICU length of stay; **(C)** hospital length of stay. MDs were calculated as higher-protein minus lower/standard-protein provision; therefore, negative values favor higher-protein provision. Random-effects inverse-variance models were used. In Figure 4B, Doig 2015 was included using the generic inverse-variance method because only the reported treatment effect was available.

Eight studies reported duration of mechanical ventilation. Higher protein provision was not associated with a statistically significant reduction in mechanical ventilation duration, with substantial heterogeneity (MD = −0.74 days, 95% CI−1.92–0.43; *I*^2^ = 79.0%; Figure 4A). Because the included trials differed in concurrent energy-delivery strategies, an exploratory subgroup analysis according to energy comparability is also presented in Supplementary Figure S1D. In trials with energy-comparable or target-comparable feeding strategies, the point estimate was lower with higher protein provision, but the confidence interval crossed the line of no effect (MD = −0.92 days, 95% CI−2.37–0.52; *I*^2^ = 87%, Supplementary Figure S1D). In trials with energy-non-comparable or caloric co-intervention strategies, no significant effect was observed (MD = −0.30 days, 95% CI−2.16–1.55; *I*^2^ = 9%, Supplementary Figure S1D). No significant subgroup difference was observed by energy-delivery strategy (*P* = 0.60, Supplementary Figure S1D). Because heterogeneity remained substantial in the energy-comparable/target-comparable subgroup, a leave-one-out influence analysis was performed to explore residual heterogeneity, as summarized in Supplementary Table S4.

Nine studies reported ICU length of stay. Higher protein provision did not significantly reduce ICU stay (MD = −0.17 days, 95% CI−1.39–1.04; *I*^2^ = 17%; Figure 4B). An exploratory subgroup analysis by energy-delivery strategy is presented in Supplementary Figure S1E. Doig 2015 was retained in the primary analysis but excluded from this subgroup analysis, as its add-on intravenous amino acid design precluded clear classification of total energy exposure. The subgroup analysis did not suggest clear effect modification by energy-delivery strategy.

Five studies reported hospital length of stay, and the pooled result also showed no significant difference between groups (MD = 1.13 days, 95% CI−3.94–6.20; *I*^2^ = 49.1%; Figure 4C). Formal subgroup analysis by energy-delivery strategy was not performed, as the energy-non-comparable and unclear/add-on categories each contained only one study.

Free-day outcomes, including ventilator-free days, ICU-free days, and hospital-free days, were not pooled because each specific endpoint was available from only one study in the current dataset.

### Muscle mass and muscle function outcomes

Muscle-related outcomes were reported across several studies but were highly heterogeneous in anatomical site, measurement method, and follow-up time point. Reported outcomes included quadriceps or rectus femoris thickness, rectus femoris cross-sectional area, biceps muscle thickness, diaphragm thickness or thickening fraction, handgrip strength, and fatigue score (Supplementary Table S5). Although some individual studies reported differences in selected muscle-related indices, the eligible studies meeting the ≥0.3 g/kg/day protein-difference criterion did not provide sufficiently comparable data for quantitative synthesis. Therefore, muscle mass and muscle function outcomes were summarized descriptively rather than pooled, and no firm conclusion could be drawn regarding the effect of higher protein provision on muscle preservation or functional recovery.

### Nutrition and metabolic outcomes

Nitrogen balance was considered the key mechanistic nutrition/metabolic outcome. Albumin was analyzed as an exploratory biochemical marker rather than a direct marker of nutritional status, because serum albumin in critical illness is strongly influenced by systemic inflammation, capillary leakage, fluid balance, and hepatic function. Blood urea nitrogen and creatinine were evaluated as laboratory indicators of protein metabolic burden and renal safety. For nutrition/metabolic and safety outcomes, formal subgroup analyses by energy-delivery strategy were not performed because most outcomes included only two studies or had fewer than two studies within one or more energy-defined subgroups.

**Figure 5:**
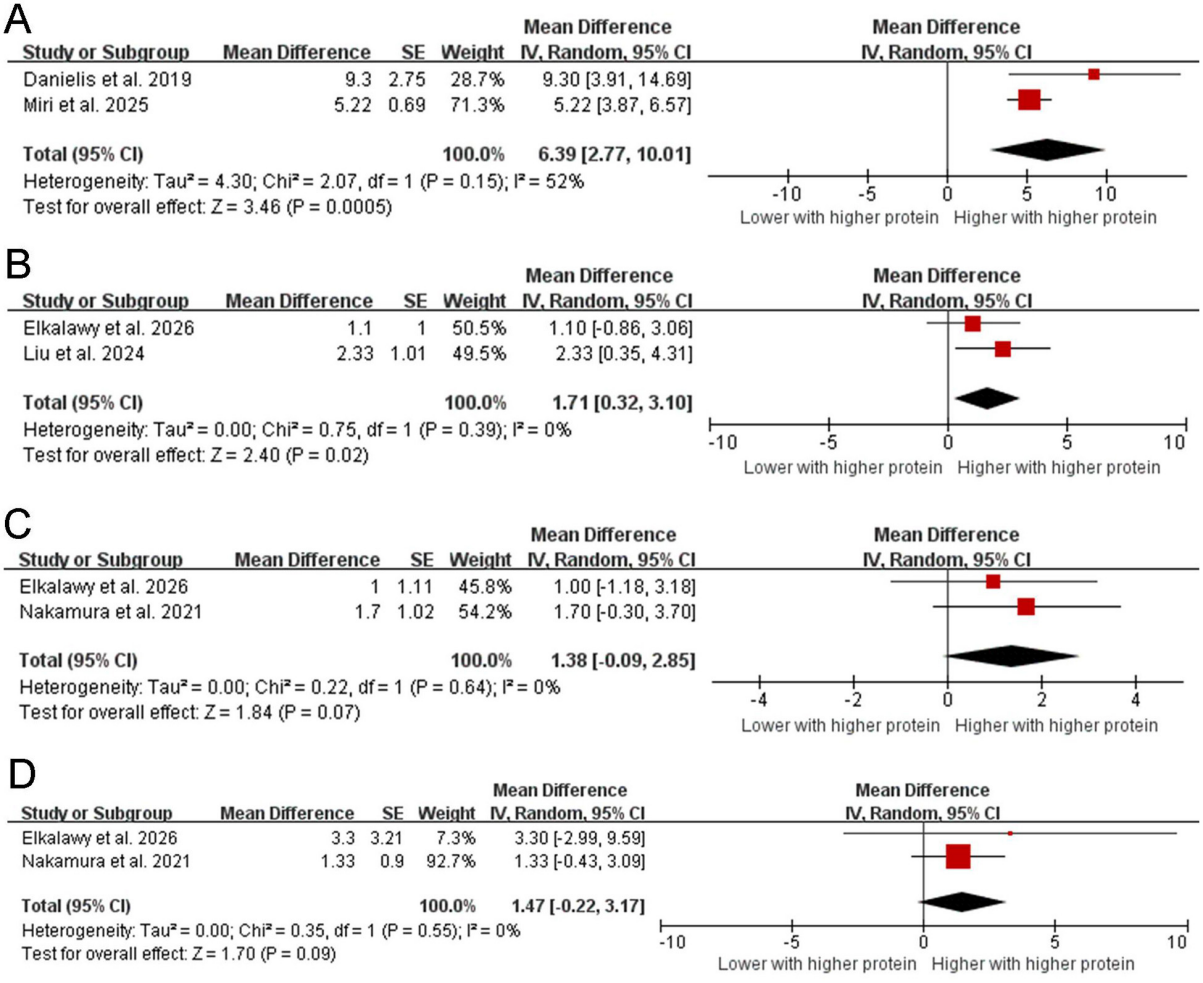
Forest plots of nutrition and metabolic outcomes. **(A)** Day 3 nitrogen balance; **(B)** Day 5 serum albumin; **(C)** Day 10 serum albumin; **(D)** Day 10 blood urea nitrogen (BUN). MDs were calculated as higher-protein minus lower/standard-protein provision; therefore, positive values indicate higher values in the higher-protein group and should not necessarily be interpreted as clinical benefit. Random-effects inverse-variance models were used. Nitrogen balance represents a mechanistic surrogate outcome, whereas serum albumin and BUN are exploratory biochemical markers and should not be interpreted as direct evidence of improved nutritional status or clinical recovery. Abbreviations: BUN, blood urea nitrogen; MD, mean difference.

Day 3 nitrogen balance was reported by two eligible studies. Compared with lower or standard protein provision, higher protein provision was associated with significantly more favorable Day 3 nitrogen balance (MD = 6.39 g/day, 95% CI 2.77–10.01; *I*^2^ = 51.7%; Figure 5A). Although this result is consistent with a measurable short-term metabolic difference, nitrogen balance represents a mechanistic surrogate outcome rather than a direct patient-centered clinical endpoint. Therefore, this finding should be interpreted cautiously given the limited number of included studies.

For Day 5 nitrogen balance, the point estimate was higher with higher protein provision, but the confidence interval was wide and crossed the line of no effect (MD = 5.84 g/day, 95% CI−2.00–13.68; *I*^2^ = 83.3%; Supplementary Figure S2A), and the finding was not considered robust. Higher protein provision was associated with higher Day 5 serum albumin concentrations (MD = 1.71 g/L, 95% CI 0.32–3.10; *I*^2^ = 0%; Figure 5B). For Day 10 serum albumin, no statistically significant difference was observed (MD = 1.38 g/L, 95% CI−0.09–2.85; *I*^2^ = 0%; Figure 5C). Given the indirect and nonspecific nature of serum albumin in critical illness, these findings were considered exploratory and should not be interpreted as evidence of improved nutritional status or protein adequacy.

For Day 10 blood urea nitrogen (BUN), no statistically significant difference was observed between groups, although the point estimate was higher with higher protein provision (MD = 1.48 mg/dL, 95% CI−0.22–3.17; *I*^2^ = 0%; Figure 5D). Creatinine could not be pooled because comparable fixed-time-point data were insufficient. Descriptive findings did not suggest a consistent signal of clinically meaningful creatinine elevation or renal deterioration with higher protein provision.

### Safety outcomes

Two studies reported a gastrointestinal intolerance composite outcome. No statistically significant difference was observed between higher and lower/standard protein provision, but heterogeneity was substantial and the confidence interval was wide (RR = 1.00, 95% CI 0.32–3.16; *I*^2^ = 74.4%; Supplementary Figure S2B). The heterogeneity was likely related to differences in outcome definitions and opposite directions of effect between the two included trials. Therefore, this pooled estimate was considered exploratory and should not be interpreted as definitive evidence of gastrointestinal safety.

Specific adverse events, including diarrhea, gastric retention or high gastric residual volume, hyperglycemia, and AKI/RRT, were not pooled formally when fewer than two studies provided comparable event data. Available single-study data did not show a consistent signal of increased hyperglycemia, gastric retention, or renal adverse events with higher protein provision, but these findings remain uncertain because of the limited number of studies.

### Sensitivity and influence analyses

Sensitivity and influence analyses were conducted for outcomes with sufficient data to assess the robustness of pooled estimates and, where relevant, to explore potential sources of heterogeneity. For 28/30-day and 90-day mortality, leave-one-out analyses did not materially change the pooled estimates or statistical conclusions. For duration of mechanical ventilation, heterogeneity remained substantial within the energy-comparable/target-comparable subgroup; therefore, a leave-one-out influence analysis was performed in this subgroup. Exclusion of Elkalawy et al. (26) reduced heterogeneity from 87% to 41%, whereas exclusion of other individual studies did not fully resolve the residual heterogeneity. All leave-one-out estimates remained statistically nonsignificant (Supplementary Table S4). For nutrition/metabolic and safety outcomes, additional formal sensitivity analyses were limited because most outcomes included only two studies; these findings were therefore interpreted cautiously.

### Threshold-based subgroup analyses

Exploratory subgroup analyses were performed according to absolute protein intake relative to the 1.2 g/kg/day threshold. Studies were categorized as double-high, double-low, or cross-threshold comparisons. To avoid overinterpretation of sparse data, formal subgroup analyses were conducted only when at least two subgroups were available and each subgroup included at least two studies.

**Figure 6:**
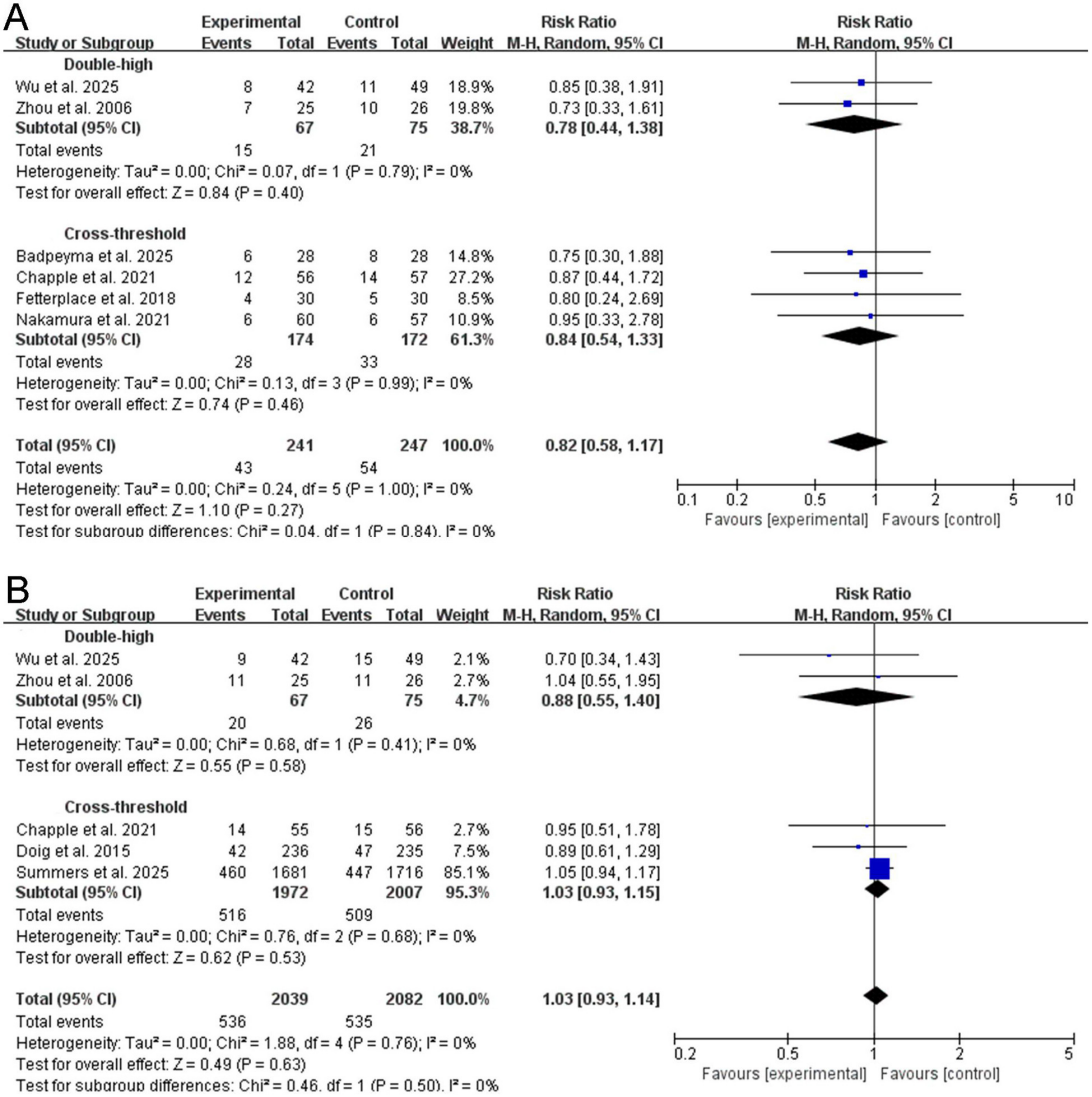
Threshold-based subgroup analyses of mortality outcomes. **(A)** 28/30-day all-cause mortality; **(B)** 90-day all-cause mortality. Subgroups were defined according to whether protein intake in the higher-protein and comparator groups was ≥1.2 g/kg/day or < 1.2 g/kg/day. Risk ratios compare higher-protein with lower/standard-protein provision; values less than 1 favor higher-protein provision.

Only 28/30-day mortality and 90-day mortality met these criteria. For 28/30-day mortality, no significant subgroup difference was observed between the double-high and cross-threshold subgroups (P for subgroup difference = 0.84; Figure 6A). Similarly, for 90-day mortality, no significant subgroup difference was observed between these subgroups (P for subgroup difference = 0.50; Figure 6B). These findings did not provide evidence that the effect of higher protein provision on mortality was modified by whether both groups achieved ≥1.2 g/kg/day or whether the comparison crossed the 1.2 g/kg/day threshold.

Other clinical recovery, nutrition/metabolic, and safety outcomes did not meet the prespecified criteria for formal threshold-based subgroup analysis and were not interpreted as subgroup effects.

### Publication bias

Formal assessment of publication bias was not performed because most outcomes included fewer than 10 studies, making funnel plot interpretation and statistical tests for small-study effects unreliable. Publication bias was considered qualitatively by examining the comprehensiveness of the search strategy and the consistency of reporting across included studies. Given the limited number of studies for most outcomes, publication bias could not be reliably excluded.

### Certainty of evidence

The certainty of evidence was assessed using the GRADE approach. The certainty of evidence was moderate for 28/30-day mortality and 90-day mortality. Evidence for 60-day mortality, in-hospital mortality, and ICU length of stay was low. Evidence for ICU mortality, duration of mechanical ventilation, hospital length of stay, Day 3 nitrogen balance, and gastrointestinal intolerance was very low.

The main reasons for downgrading were risk-of-bias concerns, inconsistency, indirectness of surrogate outcomes, small numbers of studies, wide confidence intervals, and inability to reliably assess publication bias. Overall, the certainty of evidence was limited for most outcomes, and the findings should be interpreted cautiously. The summary of findings and certainty of evidence for key outcomes are presented in Table 1.

## Discussion

### Principal findings

In this systematic review and meta-analysis of randomized controlled trials, higher protein provision with meaningful dose separation did not significantly reduce mortality in critically ill adults. No significant differences were observed for 28/30-day, 60-day, 90-day, in-hospital, or ICU mortality. Similarly, higher protein provision did not consistently improve major clinical recovery outcomes, including duration of mechanical ventilation, ICU length of stay, or hospital length of stay.

In contrast, higher protein delivery was associated with more favorable early nitrogen balance, suggesting a measurable short-term metabolic association. However, this surrogate finding did not translate into clear benefits in patient-centered outcomes. Muscle-related outcomes were reported across several trials, but differences in anatomical sites, measurement methods, and follow-up time points precluded quantitative synthesis. Safety analyses did not show a clear increase in gastrointestinal intolerance, hyperglycemia, or renal adverse events, but the available evidence was limited and imprecise.

Overall, current randomized evidence does not support a consistent benefit of higher protein provision on survival or major clinical recovery outcomes, although a more favorable early nitrogen balance was observed.

### Interpretation of findings

The absence of a significant mortality benefit suggests that protein dose alone may be insufficient to modify survival in the heterogeneous setting of critical illness. Mortality in ICU patients is strongly influenced by underlying disease severity, organ failure, inflammation, renal function, timing of nutritional intervention, and post-ICU recovery (8, 39). Thus, even a biologically plausible intervention such as higher protein delivery may not necessarily produce measurable survival benefits across broad ICU populations. Importantly, the absence of a significant mortality benefit should not be interpreted as evidence against protein therapy in general or as evidence that higher protein provision lacks clinically meaningful effects on recovery-centered outcomes, particularly muscle preservation and physical function. Rather, the neutral mortality findings suggest that the response to protein provision is likely context-dependent and may be influenced by baseline nutritional risk, metabolic status, illness phase, concurrent energy delivery, renal function, organ failure severity, and the patient's ability to utilize administered amino acids.

A key feature of this review was that mortality outcomes were analyzed separately by time point rather than pooled together. This approach improves clinical interpretability because early mortality, 90-day mortality, ICU mortality, and in-hospital mortality reflect different clinical contexts. The lower point estimate for 28/30-day mortality should therefore be interpreted cautiously, particularly because the confidence interval crossed the line of no effect and a similar pattern was not observed consistently at later time points.

For clinical recovery outcomes, higher protein provision was not associated with a statistically significant reduction in mechanical ventilation duration, and heterogeneity was substantial. Sensitivity analysis suggested that this heterogeneity was partly driven by specific studies with distinct population or design features. ICU and hospital length of stay were not significantly reduced. These outcomes are influenced by many non-nutritional factors, including discharge practices, sedation strategy, infection control, rehabilitation availability, and institutional protocols. Thus, improvements in nutritional or metabolic markers may not directly translate into shorter ICU or hospital stay.

The most consistent surrogate metabolic finding was a more favorable Day 3 nitrogen balance. This finding is biologically plausible because critically ill patients are highly catabolic, and increased amino acid delivery may partially offset nitrogen losses (39, 40). However, nitrogen balance is a surrogate outcome and should not be interpreted as direct evidence of improved clinical recovery. Similarly, serum albumin findings were exploratory because albumin is a negative acute-phase reactant and is strongly affected by systemic inflammation, capillary leakage, fluid balance, and hepatic function, rather than protein intake alone (41, 42).

Muscle preservation and functional recovery deserve particular attention when interpreting the effects of higher protein provision in critical illness. Mortality is an important outcome, but it may be relatively insensitive to the potential benefits of nutrition therapy, especially when the intervention is intended to preserve lean body mass, attenuate ICU-acquired weakness, and support recovery rather than directly modify survival. Several included trials reported muscle-related or functional outcomes, including muscle thickness, cross-sectional area, handgrip strength, fatigue, or functional recovery measures. However, the current evidence was insufficient for quantitative synthesis because the included trials assessed different anatomical sites, including quadriceps, rectus femoris, biceps, femoral muscle, and diaphragm, using different techniques, time points, and reporting formats (4, 5). Therefore, the absence of a clear mortality benefit should not be interpreted as evidence of absence of clinically relevant benefit in muscle preservation or functional recovery. Rather, the available evidence remains insufficient to determine whether higher protein delivery improves patient-centered functional outcomes. Future trials should standardize muscle and functional assessments and prioritize recovery-centered outcomes, including ICU-acquired weakness, MRC score, handgrip strength, ventilator-free days, physical function, activities of daily living, health-related quality of life, and longer-term post-discharge recovery.

Safety findings should also be interpreted cautiously. The gastrointestinal intolerance composite outcome showed no statistically significant difference, but heterogeneity was substantial and the confidence interval was wide. Specific safety outcomes, including hyperglycemia, gastric retention, AKI, and kidney replacement therapy, were generally reported by single studies and could not be pooled. Therefore, the available evidence does not show a clear safety signal, but it is insufficient to exclude potential harm in specific patient groups.

### Threshold-based subgroup analysis and comparison with previous reviews

This review attempted to classify studies according to the absolute protein intake threshold of 1.2 g/kg/day, a cutoff consistent with commonly used guideline-based targets and prior evidence syntheses (39). Formal subgroup analyses were performed only when at least two subgroups were available and each subgroup contained at least two studies. Only 28/30-day mortality and 90-day mortality met these criteria, and neither showed a significant subgroup difference. These findings do not provide evidence that mortality effects were modified by whether both groups received ≥1.2 g/kg/day or whether the comparison crossed the 1.2 g/kg/day threshold.

Previous meta-analyses have reported inconsistent or inconclusive findings regarding higher versus lower protein provision in critically ill patients (10, 43, 44). Compared with previous reviews, this study has several methodological refinements. First, the main analysis focused on trials with a between-group protein difference of at least 0.3 g/kg/day, reducing dilution from studies with minimal protein separation. Second, mortality outcomes were analyzed separately by time point. Third, subgroup analyses were based on absolute protein intake relative to the 1.2 g/kg/day threshold. Fourth, clinical recovery, nutrition/metabolic, muscle-related, and safety outcomes were interpreted separately rather than as a single group of secondary outcomes. These choices improved clinical interpretability but also highlighted the limitations of the available evidence.

Our findings should be interpreted in the context of contemporary critical care nutrition guidelines and recent landmark trials. Although ESPEN and ASPEN recommendations generally support higher protein provision in critically ill adults, both the optimal dose and timing remain uncertain, and individualized prescription is increasingly emphasized. Recent large trials, including NUTRIREA-3, EFFORT Protein, PRECISe, and TARGET Protein, have not consistently shown that routine escalation of protein delivery improves survival or patient-centered recovery in unselected ICU populations. Therefore, our findings do not support a universal “more protein is better” strategy, but rather suggest that protein therapy should be individualized according to nutritional risk, illness phase, baseline muscle reserve, renal function, organ failure severity, body composition, rehabilitation exposure, and ICU admission etiology.

### Clinical and research implications

Building on this interpretation, the main clinical implication is that protein prescription should move from a uniform target-based approach toward an individualized, phenotype- and phase-informed strategy. The neutral overall estimates do not justify routine escalation of protein provision in unselected critically ill adults, but they also do not argue against protein therapy in general or exclude benefit in selected patients.

Several patient groups may remain plausible candidates for targeted higher-protein strategies. Trauma and surgical ICU patients may have increased protein requirements because of tissue injury, surgical stress, wound healing, inflammation, and hypercatabolism (45, 46). Older adults, frail patients, and patients with sarcopenia may have lower baseline muscle reserve and greater vulnerability to ICU-acquired weakness, making muscle preservation and functional recovery particularly relevant outcomes (47). Obese critically ill patients may represent another distinct subgroup in whom hypocaloric high-protein feeding could theoretically preserve lean body mass while reducing the risks of overfeeding (48). In addition, patients entering the recovery or persistent critical illness phase may be more capable of utilizing exogenous protein than patients in the earliest acute phase of critical illness.

Baseline nutritional risk may be another key modifier of response to higher protein provision. Patients with high nutritional risk, pre-existing malnutrition, low muscle reserve, or prolonged inadequate intake may be more likely to benefit from optimized protein delivery than well-nourished, nutritionally low-risk patients. Conversely, applying the same higher-protein target to nutritionally low-risk patients may dilute potential benefit and expose many patients to an intervention from which they are unlikely to derive clinically meaningful improvement. Contemporary critical care nutrition increasingly emphasizes individualized nutrition therapy rather than uniform macronutrient targets for all ICU patients. Future trials should therefore stratify patients by validated nutritional risk tools, such as the NUTRIC or modified NUTRIC score, and report whether treatment effects differ according to baseline nutritional risk, malnutrition status, body composition, and duration of inadequate intake (49, 50).

The phase of critical illness may therefore be an important determinant of response to higher protein provision. Protein delivered during the early acute phase, when patients often have marked inflammation, insulin resistance, impaired substrate utilization, and ongoing organ dysfunction, may have different physiological effects from protein delivered during the recovery or persistent critical illness phase. Recent trials further highlight this uncertainty. NUTRIREA-3 suggested potential benefit from restricting both calorie and protein intake during the early phase of shock (51), whereas EFFORT Protein, PRECISe, and TARGET Protein did not demonstrate consistent patient-centered benefit from higher protein delivery during critical illness (16, 27, 29). These findings support the concept that protein prescription should be both phenotype-specific and phase-specific rather than applied uniformly throughout the ICU stay.

Importantly, the absence of a clear safety signal should not be used as a rationale for universal higher-protein feeding in all critically ill patients. Instead, our findings support a targeted and individualized approach, in which higher protein targets are considered for biologically plausible candidate populations while accounting for nutritional risk, illness phase, baseline muscle status, body composition, renal function, organ failure severity, feeding tolerance, energy-delivery strategy, and rehabilitation goals.

These hypotheses remain insufficiently tested in the current randomized evidence because most trials enrolled mixed ICU populations and did not report subgroup-specific treatment effects. Future trials should prespecify clinically relevant subgroups, including trauma or surgical ICU patients, older adults, patients with sarcopenia or frailty, patients with obesity, and patients in the recovery or persistent critical illness phase. Trials should distinguish early acute, late acute, and recovery phases; report actual delivered protein intake, energy intake, renal safety outcomes, body composition, rehabilitation exposure, and standardized functional outcomes; and test whether delayed escalation or phase-adapted protein delivery improves muscle, functional, and patient-centered outcomes. Because protein alone may be insufficient to improve long-term recovery, future interventions should consider combining optimized protein delivery with strategies targeting muscle preservation and functional recovery.

### Strengths and limitations

This review has several strengths. It included only randomized controlled trials, focused on clinically meaningful protein dose separation, analyzed mortality by prespecified time points, assessed a broad range of patient-centered and mechanistic outcomes, applied conservative criteria for subgroup analyses, and evaluated certainty of evidence using the GRADE approach.

Several limitations should be acknowledged. First, the included trials were clinically heterogeneous, with differences in ICU admission etiology, disease severity, intervention strategies, timing of protein delivery, concurrent energy intake, and achieved protein exposure. The enrolled populations included mixed mechanically ventilated ICU patients as well as more specific cohorts such as trauma or surgical ICU patients, traumatic brain injury, sepsis, severe pneumonia, severe stroke, and prolonged mechanical ventilation. These populations may differ substantially in metabolic response to critical illness, inflammatory burden, tissue injury, protein catabolism, protein requirements, rehabilitation potential, and expected response to nutritional interventions. Although ICU admission etiology and case mix were extracted when reported and summarized in Table 2 and Supplementary Table S3, formal subgroup analysis by admission etiology was not feasible because many trials enrolled mixed ICU populations and did not report etiology-specific treatment effects. Therefore, the pooled estimates should be interpreted as average effects across heterogeneous adult critically ill populations and should not be considered definitive evidence excluding benefit or harm in specific subgroups, such as trauma, surgical, neurological, septic, or prolonged mechanical ventilation patients.

Second, we could not reliably evaluate the influence of illness phase. Most included trials initiated nutrition interventions during the early ICU period and did not report treatment effects separately for the early acute, late acute, recovery, or persistent critical illness phases. Therefore, the present analysis cannot determine whether higher protein provision is more beneficial or less harmful when initiated after initial stabilization or during recovery rather than during the earliest acute phase.

Third, actual delivered protein and energy intake were not consistently available, and some exposure classifications relied on prescribed targets or converted estimates. This limitation is particularly relevant because higher-protein feeding may have different implications when delivered under isocaloric, hypocaloric–hyperproteic, or higher-energy co-intervention paradigms. In addition, the intervention contrast could not always be interpreted as “protein alone.” Several trials differed simultaneously in caloric intake, protein-to-energy ratio, formula composition, amino acid supplementation, feeding route, or broader nutrition-delivery strategy. Therefore, the observed effects cannot be attributed solely to protein dose with complete confidence, and residual confounding by nutrition-related co-interventions may remain. Although we extracted energy delivery and feeding paradigm and performed exploratory subgroup or sensitivity analyses where feasible, incomplete reporting and sparse outcome data limited our ability to fully isolate the independent effect of protein provision. The 0.3 g/kg/day threshold was also a pragmatic analytic criterion rather than an established biological cutoff. Although it was used to ensure meaningful between-group protein separation and reduce exposure misclassification, alternative thresholds could yield different study inclusion and pooled estimates.

Fourth, age and baseline nutritional risk could not be adequately evaluated as effect modifiers. Older adults, sarcopenic patients, and nutritionally high-risk patients may be more vulnerable to muscle wasting and may represent subgroups more likely to benefit from optimized protein delivery. Although mean or median age was extracted where available, formal age-based subgroup analysis was not feasible because most trials did not report age-stratified outcomes, and study-level mean age could not reliably identify older or sarcopenic patients. Similarly, most studies did not report treatment effects stratified by NUTRIC score, modified NUTRIC score, malnutrition status, or baseline muscle reserve. Therefore, potential differential effects in older adults, sarcopenic patients, or nutritionally high-risk populations could not be assessed.

Fifth, this review included only adult critically ill patients, and no pediatric ICU trials were eligible for inclusion. Therefore, the findings should not be extrapolated to pediatric critical care populations, in whom protein requirements, growth-related nutritional needs, metabolism, disease patterns, and tolerance of nutrition support may differ substantially from those of adults.

Sixth, several continuous outcomes required conversion from medians and interquartile ranges, which may introduce uncertainty. Many secondary outcomes, including nutrition/metabolic, muscle-related, and safety outcomes, were reported by few studies and were therefore exploratory. Publication bias could not be reliably assessed for most outcomes because fewer than 10 studies were available. Finally, the certainty of evidence was low or very low for many outcomes, mainly because of risk-of-bias concerns, inconsistency, indirectness, and imprecision.

## Conclusions

In critically ill adults, higher protein provision with meaningful dose separation did not significantly reduce mortality or consistently improve clinical recovery outcomes compared with lower or standard protein provision. Higher protein provision was associated with more favorable early nitrogen balance, but this finding represents a surrogate metabolic outcome and does not establish improved clinical recovery. Evidence was insufficient to determine its effect on muscle mass or muscle function. No clear increase in gastrointestinal intolerance or kidney-related safety events was observed, although safety evidence was limited.

The certainty of evidence was low or very low for many outcomes. Current evidence therefore does not support a universal high-protein strategy for all critically ill patients, but suggests that protein provision should be individualized and studied further in well-designed randomized trials with standardized protein targets, actual delivery reporting, safety monitoring, and patient-centered outcomes.

## Data Availability

The original contributions presented in the study are included in the article/supplementary material, further inquiries can be directed to the corresponding author.

## References

[1] Preiser JC Ichai C Orban JC Groeneveld AB. Metabolic response to the stress of critical illness. Br J Anaesth. (2014) 113:945–54. doi: 10.1093/bja/aeu187 24970271

[2] Rousseau AF Martindale R. Nutritional and metabolic modulation of inflammation in critically ill patients: a narrative review of rationale, evidence and grey areas. Ann Intensive Care. (2024) 14:121. doi: 10.1186/s13613-024-01350-x 39088114 PMC11294317

[3] Kang MC. Muscle Protein Metabolism in Critically Illness. Ann Clin Nutr Metab. (2020) 11:35–9. doi: 10.18858/smn.2020.11.2.35

[4] Puthucheary ZA Rawal J McPhail M Connolly B Ratnayake G Chan P . Acute skeletal muscle wasting in critical illness. JAMA. (2013) 310:1591–600. doi: 10.1001/jama.2013.278481 24108501

[5] Fazzini B Märkl T Costas C Blobner M Schaller SJ Prowle J . The rate and assessment of muscle wasting during critical illness: a systematic review and meta-analysis. Crit Care. (2023) 27:2. doi: 10.1186/s13054-022-04253-0 36597123 PMC9808763

[6] De Jonghe B Sharshar T Lefaucheur JP Authier FJ Durand-Zaleski I Boussarsar M . Paresis acquired in the intensive care unit: a prospective multicenter study. JAMA. (2002) 288:2859–67. doi: 10.1001/jama.288.22.2859 12472328

[7] Singer P Blaser AR Berger MM Calder PC Casaer M Hiesmayr M . ESPEN practical and partially revised guideline: Clinical nutrition in the intensive care unit. Clin Nutr. (2023) 42:1671–89. doi: 10.1016/j.clnu.2023.07.011 37517372

[8] Compher C Bingham AL McCall M Patel J Rice TW Baraunschweig C . Guidelines for the provision of nutrition support therapy in the adult critically ill patient: The American Society for Parenteral and Enteral Nutrition. JPEN J Parenter Enteral Nutr. (2022) 46:12–41. doi: 10.1002/jpen.2267 34784064

[9] Bels JLM van Gassel RJJ van de Poll MCG. Protein supplementation in critical illness: why, when and how? Curr Opin Clin Nutr Metab Care. (2023) 26:145–52. doi: 10.1097/MCO.0000000000000912 36728596

[10] Lee ZY Dresen E Lew CCH Bels J Hill A Hasan MS . The effects of higher versus lower protein delivery in critically ill patients: an updated systematic review and meta-analysis of randomized controlled trials with trial sequential analysis. Crit Care. (2024) 28:15. doi: 10.1186/s13054-023-04783-1 38184658 PMC10770947

[11] McKenzie JE Brennan SE Ryan RE Thomson HJ Johnston RV Thomas J. Defining the criteria for including studies and how they will be grouped for the synthesis. In: Higgins JPT Thomas J Chandler J Cumpston M Li T Page MJ Welch VA, eds. Cochrane Handbook for Systematic Reviews of Interventions. Version 6.5. Cochrane; 2024. Chapter 3.

[12] Wan X Wang W Liu J Tong T. Estimating the sample mean and standard deviation from the sample size, median, range and/or interquartile range. BMC Med Res Methodol. (2014) 14:135. doi: 10.1186/1471-2288-14-135 25524443 PMC4383202

[13] Arabi YM Al-Dorzi HM Tamim H Sadat M Al-Hameed F AlGhamdi A . Replacing protein via enteral nutrition in a stepwise approach in critically ill patients: a pilot randomized controlled trial (REPLENISH pilot trial). Clin Nutr ESPEN. (2021) 44:166–72. doi: 10.1016/j.clnesp.2021.05.008 34330462

[14] Azevedo JRA Lima HCM Montenegro WS Souza SCC Nogueira IROM Silva MM . Optimized calorie and high protein intake versus recommended caloric-protein intake in critically ill patients: a prospective, randomized, controlled phase II clinical trial. Rev Bras Ter Intensiva. (2019) 31:171–9. doi: 10.5935/0103-507X.20190025 31141081 PMC6649219

[15] Badpeyma M Sedaghat A Moghaddam AB Khadem-Rezaiyan M Sistanian F Bagherniya M . The efficacy of high-protein nutritional support on mortality, clinical outcomes, and nutritional adequacy in critically ill patients: a double-center randomized controlled trial. Nutr Metab. (2025) 22:116. doi: 10.1186/s12986-025-01003-1 41063248 PMC12506466

[16] Bels JLM Thiessen S van Gassel RJJ Beishuizen A De Bie Dekker A Fraipont V . Effect of high versus standard protein provision on functional recovery in people with critical illness (PRECISe): an investigator-initiated, double-blinded, multicentre, parallel-group, randomised controlled trial in Belgium and the Netherlands. Lancet. (2024) 404:659–69. doi: 10.1016/S0140-6736(24)01304-7 39153816

[17] Chapple LS Summers MJ Bellomo R Chapman MJ Davies AR Ferrie S . Use of a high-protein enteral nutrition formula to increase protein delivery to critically ill patients: a randomized, blinded, parallel-group, feasibility trial. JPEN J Parenter Enteral Nutr. (2021) 45:699–709. doi: 10.1002/jpen.2059 33296079

[18] Liu C He L Zhang JH He J Tian L Zheng X. Impact of high-protein enteral nutrition on muscle preservation in mechanically ventilated patients with severe pneumonia: a randomized controlled trial. J Health Popul Nutr. (2024) 43:152. doi: 10.1186/s41043-024-00633-0 39342405 PMC11439213

[19] Hepduman D Sungurtekin H. Effect of high or low protein nutrition on diaphragm thickness using ultrasonography in mechanically ventilated intensive care patients. Clin Sci Nutr. (2025) 7:47–54. doi: 10.62210/ClinSciNutr.2025.103

[20] Doig GS Simpson F Bellomo R Heighes PT Sweetman EA Chesher D . Intravenous amino acid therapy for kidney function in critically ill patients: a randomized controlled trial. Intensive Care Med. (2015) 41:1197–208. doi: 10.1007/s00134-015-3827-9 25925203

[21] Dresen E Weißbrich C Fimmers R Putensen C Stehle P. Medical high-protein nutrition therapy and loss of muscle mass in adult ICU patients: a randomized controlled trial. Clin Nutr. (2021) 40:1562–70. doi: 10.1016/j.clnu.2021.02.021 33743292

[22] Uyar E Yagmurdur H Yamanyar S Güdek Y Dal MC Cosar A. The effect of protein enriched nutrition on diaphragm function in mechanically ventilated patients. Nutr Clin Metab. (2023) 37:31–8. doi: 10.1016/j.nupar.2022.10.001

[23] Fetterplace K Deane AM Tierney A Beach LJ Knight LD Presneill J . Targeted full energy and protein delivery in critically ill patients: a pilot randomized controlled trial (FEED trial). JPEN J Parenter Enteral Nutr. (2018) 42:1252–62. doi: 10.1002/jpen.1166 29701878

[24] García-de-Lorenzo A Ortíz-Leyba C Planas M Montejo JC Núñez R Ordóñez FJ . Parenteral administration of different amounts of branch-chain amino acids in septic patients: clinical and metabolic aspects. Crit Care Med. (1997) 25:418-424. doi: 10.1097/00003246-199703000-00008 9118656

[25] O'Keefe GE Brown SP Shelton MM Qiu Q Bisgaard EK Wilson IM . Enteral protein supplementation in critically ill trauma and surgical patients: a single-center randomized clinical trial. J Trauma Acute Care Surg. (2025) 99:635–42. doi: 10.1097/TA.0000000000004745 40671181

[26] Elkalawy H Sekhar P Fayad M Barrima M Abdullah M. Effect of standard- versus high-protein enteral feeding on rectus femoris muscle mass in mechanically ventilated traumatic brain injury patients: a prospective randomized study in Egypt and the United States. Acute Crit Care. (2026) 41:160–73. doi: 10.4266/acc.001025 41457760 PMC12989931

[27] Heyland DK Patel J Compher C Rice TW Bear DE Lee ZY . The effect of higher protein dosing in critically ill patients with high nutritional risk (EFFORT Protein): an international, multicentre, pragmatic, registry-based randomised trial. Lancet. (2023) 401:568–76. doi: 10.1016/S0140-6736(22)02469-2 36708732

[28] Danielis M Lorenzoni G Azzolina D Iacobucci A Trombini O De Monte A . Effect of protein-fortified diet on nitrogen balance in critically ill patients: results from the OPINiB trial. Nutrients. (2019) 11:972. doi: 10.3390/nu11050972 31035354 PMC6567073

[29] Summers MJ Chapple LS Deane AM. Augmented enteral protein during critical illness: the TARGET Protein randomized clinical trial. JAMA. (2025) 334:319–28. doi: 10.1001/jama.2025.13868 40495743 PMC12159859

[30] Miri MM Kouchek M Salarian S Sistanizad M Sorbi T Eslamian R . Impact of high-protein enteral feeding on skeletal muscle mass changes in critically ill patients: a randomized controlled trial. Med J Islam Repub Iran. (2025) 39:114. doi: 10.47176/mjiri.39.114 41194956 PMC12584090

[31] Nakamura K Nakano H Naraba H Mochizuki M Takahashi Y Sonoo T . High protein versus medium protein delivery under equal total energy delivery in critical care: a randomized controlled trial. Clin Nutr. (2021) 40:796–803. doi: 10.1016/j.clnu.2020.07.036 32800385

[32] Rugeles SJ Rueda JD Díaz CE Rosselli D. Hyperproteic hypocaloric enteral nutrition in the critically ill patient: a randomized controlled clinical trial. Indian J Crit Care Med. (2013) 17:343–9. doi: 10.4103/0972-5229.123438 24501485 PMC3902568

[33] Jakob SM Bütikofer L Berger D Coslovsky M Takala J. A randomized controlled pilot study to evaluate the effect of an enteral formulation designed to improve gastrointestinal tolerance in the critically ill patient—the SPIRIT trial. Crit Care. (2017) 21:140. doi: 10.1186/s13054-017-1730-1 28599662 PMC5466775

[34] van Zanten ARH Petit L De Waele J Kieft H de Wilde J van Horssen P . Very high intact-protein formula successfully provides protein intake according to nutritional recommendations in overweight critically ill patients: a double-blind randomized trial. Crit Care. (2018) 22:156. doi: 10.1186/s13054-018-2070-5 29895309 PMC5998555

[35] Parasuraman V Anand RK Khanna P Shanmugam R Ray BR. Effect of high protein normocaloric nutrition on skeletal muscle wasting in critically ill mechanically ventilated patients: a randomized double-blind study. Indian J Crit Care Med. (2025) 29:431–40. doi: 10.5005/jp-journals-10071-24966 40416531 PMC12101975

[36] Wu W Leng F Dong M Song J Zhang J Han F . Efficacy and safety of high protein intake in critically ill patients. Chin Med J (Engl). (2025) 138:880–2. doi: 10.1097/CM9.0000000000003528 40008786 PMC11970806

[37] Zhang Q Zhou J Zhu D Zhou S. Evaluation of the effect of high protein supply on diaphragm atrophy in critically ill patients receiving prolonged mechanical ventilation. Nutr Clin Pract. (2022) 37:402–12. doi: 10.1002/ncp.10672 34101252

[38] Zhou CP Su Y. Effect of the equal non-protein-calorie but different protein intake on enteral nutritional metabolism in 51 patients with severe stroke: a randomized controlled study. Chin J Clin Nutr. (2006) 14:351–5.

[39] Singer P Blaser AR Berger MM Alhazzani W Calder PC Casaer MP . ESPEN guideline on clinical nutrition in the intensive care unit. Clin Nutr. (2019) 38:48–79. doi: 10.1016/j.clnu.2018.08.037 30348463

[40] Zhu YB Yao Y Xu Y Huang HB. Nitrogen balance and outcomes in critically ill patients: a systematic review and meta-analysis. Front Nutr. (2022) 9:961207. doi: 10.3389/fnut.2022.961207 36071933 PMC9441883

[41] Yeh DD Johnson E Harrison T Kaafarani HMA Lee J Fagenholz P . Serum levels of albumin and prealbumin do not correlate with nutrient delivery in surgical intensive care unit patients. Nutr Clin Pract. (2018) 33:419–25. doi: 10.1002/ncp.10087 29665145

[42] Evans DC Corkins MR Malone A Miller S Mogensen KM Guenter P . ASPEN Malnutrition Committee. The use of visceral proteins as nutrition markers: an ASPEN position paper. Nutr Clin Pract. (2021) 36:22–8. doi: 10.1002/ncp.10588 33125793

[43] Qin Y Huang J Ping X Zheng H Zhang K Xu X . No benefit of higher protein dosing in critically ill patients: a systematic review and meta-analysis of randomized controlled trials. PeerJ. (2024) 12:e17433. doi: 10.7717/peerj.17433 38799065 PMC11122048

[44] Blaauw L Schoonees A Robertson N Visser J. The impact of guideline recommended protein intake on mortality and length of intensive care unit and hospital stay in critically ill adults: a systematic review. Clin Nutr ESPEN. (2024) 61:356–68. doi: 10.1016/j.clnesp.2024.04.003 38777455

[45] Hartwell JL Evans DC Martin MJ. Nutritional support for the trauma and emergency general surgery patient: What you need to know. J Trauma Acute Care Surg. (2024) 96:855–64. doi: 10.1097/TA.0000000000004283 38409684

[46] Hasenboehler E Williams A Leinhase I Morgan SJ Smith WR Moore EE . Metabolic changes after polytrauma: an imperative for early nutritional support. World J Emerg Surg. (2006) 1:29. doi: 10.1186/1749-7922-1-29 17020610 PMC1594568

[47] van der Steen-Dieperink MJMM Koekkoek WAC Kouw IWK. Sarcopenia and frailty in critical illness. Curr Opin Clin Nutr Metab Care. (2025) 28:192–9. doi: 10.1097/MCO.0000000000001123 40072495 PMC11970596

[48] Dickerson RN. Hypocaloric, high-protein nutrition therapy for critically ill patients with obesity. Nutr Clin Pract. (2014) 29:786–91. doi: 10.1177/0884533614542439 25049263

[49] Heyland DK Dhaliwal R Jiang X Day AG. Identifying critically ill patients who benefit the most from nutrition therapy: the development and initial validation of a novel risk assessment tool. Crit Care. (2011) 15:R268. doi: 10.1186/cc10546 22085763 PMC3388687

[50] Rahman A Hasan RM Agarwala R Martin C Day AG Heyland DK. Identifying critically-ill patients who will benefit most from nutritional therapy: further validation of the “modified NUTRIC” nutritional risk assessment tool. Clin Nutr. (2016) 35:158–62. doi: 10.1016/j.clnu.2015.01.015 25698099

[51] Reignier J Plantefeve G Mira JP Argaud L Asfar P Aissaoui N . Low versus standard calorie and protein feeding in ventilated adults with shock: a randomised, controlled, multicentre, open-label, parallel-group trial (NUTRIREA-3). Lancet Respir Med. (2023) 11:602–12. doi: 10.1016/S2213-2600(23)00092-9 36958363

