# Supplementary material for: Efficacy and safety of higher versus lower protein provision with meaningful dose separation in critically ill adults: a systematic review and meta-analysis of randomized controlled trials
Source: Front Nutr. 2026 Jul 31;13:1893988. doi: 10.3389/fnut.2026.1893988 (PMC13472815; doi:10.3389/fnut.2026.1893988)
Supplement: Supplementary file 1 [file Table_1.docx]

| **Database** | **Search strategy** |
| --- | --- |
| **Ovid Embase** | 1. Intensive Care Units/ 2. Critical Illness/ 3. Critical Care/ 4. (ICU or "intensive care" or "critical care" or "critical ill*" or "critically ill*").ti,ab. 5. 1 or 2 or 3 or 4 6. Dietary Proteins/ 7. Enteral Nutrition/ 8. Parenteral Nutrition/ 9. ("protein intake" or "protein provision" or "protein delivery" or "protein supplementation" or "protein feeding").ti,ab. 10. ("high protein" or "higher protein" or "increased protein" or "protein dose" or "protein dosing").ti,ab. 11. ("amino acid*" or "nitrogen balance").ti,ab. 12. 6 or 7 or 8 or 9 or 10 or 11 13. Mortality/ 14. Hospital Mortality/ 15. (mortalit* or survival or death or "length of stay" or "mechanical ventilation" or "muscle mass").ti,ab. 16. 13 or 14 or 15 17. Randomized Controlled Trial/ 18. Controlled Clinical Trial/ 19. (randomized or randomised or RCT or "controlled trial" or placebo).ti,ab. 20. 17 or 18 or 19 21. ("1.2 g/kg" or "1.5 g/kg" or "2.0 g/kg" or "high protein" or "higher protein" or "protein dose*").ti,ab. 22. 5 and 12 and 16 and 20 and 21 |
| **PubMed** | ("high protein"[tiab] OR "high-protein"[tiab] OR "protein supplementation"[tiab] OR "protein intake"[tiab] OR "protein dose"[tiab] OR "protein delivery"[tiab]) AND ("critical illness"[tiab] OR "critically ill"[tiab] OR "intensive care"[tiab] OR ICU[tiab]) AND (mortality[tiab] OR outcome[tiab] OR survival[tiab]) AND (randomized controlled trial[pt] OR randomised[tiab]) |
| **Cochrane Library** | (ICU OR "intensive care" OR "critical care" OR "critically ill*" OR "critical illness") AND ("high protein" OR "higher protein" OR "protein dose*" OR "protein provision" OR "protein intake" OR "protein delivery") AND (mortalit* OR survival OR "length of stay" OR "mechanical ventilation") |
| **China National Knowledge Infrastructure (CNKI)** | ("重症" OR "危重症" OR "重症监护" OR "重症监护室" OR "ICU" OR "重症患者" OR "危重患者") AND ("高蛋白" OR "蛋白补充" OR "蛋白摄入" OR "蛋白供给" OR "蛋白质摄入" OR "蛋白质供给" OR "氨基酸" OR "氮平衡" OR "肠内营养" OR "肠外营养") AND ("死亡率" OR "预后" OR "生存" OR "住院时间" OR "机械通气" OR "肌肉量" OR "肌肉厚度") AND ("随机" OR "随机对照" OR "随机对照试验" OR "RCT") |

**Supplementary Table S1. Search strategies used for each database.** Search limits: from database inception to May 1, 2026, with no restrictions on language or publication date.

| **Outcome** | **Studies** | **Participants** | **Study design** | **Risk of bias** | **Inconsistency** | **Indirectness** | **Imprecision** | **Publication bias** | **Effect estimate** | **Certainty** | **GRADE symbols** | **Importance** | **Downgrade rationale** |
| --- | --- | --- | --- | --- | --- | --- | --- | --- | --- | --- | --- | --- | --- |
| 28/30-day all-cause mortality | 6 | 488 | Randomized trials | Not serious | Not serious | Not serious | Serious | Not assessable | RR 0.82 (95% CI 0.58 to 1.17) | Moderate | ⊕⊕⊕◯ | Critical | Downgraded one level for imprecision because the 95% CI crossed the line of no effect. |
| 60-day all-cause mortality | 3 | 1406 | Randomized trials | Not serious | Serious | Not serious | Serious | Not assessable | RR 0.85 (95% CI 0.52 to 1.39) | Low | ⊕⊕◯◯ | Critical | Downgraded for moderate heterogeneity and a wide confidence interval crossing the line of no effect. |
| 90-day all-cause mortality | 5 | 4121 | Randomized trials | Serious | Not serious | Not serious | Not serious | Not assessable | RR 1.03 (95% CI 0.93 to 1.14) | Moderate | ⊕⊕⊕◯ | Critical | Downgraded one level for some concerns in risk of bias across included trials; the estimate was precise and heterogeneity was low. |
| In-hospital mortality | 2 | 620 | Randomized trials | Serious | Not serious | Not serious | Serious | Not assessable | RR 1.04 (95% CI 0.78 to 1.39) | Low | ⊕⊕◯◯ | Critical | Downgraded for risk-of-bias concerns and imprecision due to few studies and confidence interval crossing no effect. |
| ICU mortality | 2 | 161 | Randomized trials | Serious | Not serious | Not serious | Very serious | Not assessable | RR 0.84 (95% CI 0.57 to 1.26) | Very low | ⊕◯◯◯ | Critical | Downgraded for risk-of-bias concerns and very serious imprecision due to a small sample size and wide confidence interval. |
| Duration of mechanical ventilation | 8 | 3973 | Randomized trials | Serious | Very serious | Not serious | Serious | Not assessable | MD -0.74 days (95% CI -1.92 to 0.43) | Very low | ⊕◯◯◯ | Important | Downgraded for risk-of-bias concerns, high heterogeneity, and imprecision because the confidence interval crossed no effect. |
| ICU length of stay | 9 | 1163 | Randomized trials | Serious | Not serious | Not serious | Serious | Not assessable | MD -0.17 days (95% CI -1.39 to 1.04) | Low | ⊕⊕◯◯ | Important | Downgraded for risk-of-bias concerns and imprecision because the confidence interval crossed no effect. |
| Hospital length of stay | 5 | 820 | Randomized trials | Serious | Serious | Not serious | Very serious | Not assessable | MD 1.13 days (95% CI -3.94 to 6.20) | Very low | ⊕◯◯◯ | Important | Downgraded for risk-of-bias concerns, heterogeneity, and very serious imprecision due to a wide confidence interval. |
| Day 3 nitrogen balance | 2 | 140 | Randomized trials | Serious | Serious | Serious | Serious | Not assessable | MD 6.39 g/day (95% CI 2.77 to 10.01) | Very low | ⊕◯◯◯ | Important | Downgraded for risk-of-bias concerns, moderate heterogeneity, indirectness because nitrogen balance is a surrogate/mechanistic outcome, and few studies. |
| Gastrointestinal intolerance composite | 2 | 990 | Randomized trials | Serious | Serious | Serious | Very serious | Not assessable | RR 1.00 (95% CI 0.32 to 3.16) | Very low | ⊕◯◯◯ | Important | Downgraded for risk-of-bias concerns, heterogeneity, varying definitions of gastrointestinal intolerance, and very wide confidence interval. |

**Supplementary Table S2. Complete GRADE evidence profile for key outcomes.**

| **Study** | **Detailed admission etiology / case mix** | **Energy strategy: higher-protein arm** | **Energy strategy: comparator arm** | **Feeding paradigm / implication** |
| --- | --- | --- | --- | --- |
| Arabi et al. 2021 | Admission category: medical 14 vs 12; surgical 0 vs 0; non-operative trauma 5 vs 9. Sepsis at admission: 8 vs 8. | Permissive-underfeeding energy target: 40-60% of estimated energy expenditure; supplemental enteral protein adjusted to reach higher protein target. | Same permissive-underfeeding energy target: 40-60% of estimated energy expenditure; standard protein target. | Energy-comparable permissive underfeeding high-protein strategy. Etiology-specific outcome data were generally not available for formal subgroup pooling. |
| Azevedo et al. 2019 | Admission category: medical 46 vs 46; surgical 11 vs 17. Primary ICU diagnosis: cardiovascular 17 vs 23; respiratory 9 vs 11; neurological 11 vs 9; gastrointestinal 4 vs 4; sepsis 12 vs 15; other 4 vs 1. | Optimized calorie strategy; caloric intake determined by indirect calorimetry; high protein target 2.0-2.2 g/kg/day. | Recommended caloric-protein intake; 25 kcal/kg/day with protein target 1.4-1.5 g/kg/day. | Energy-comparable high-protein strategy; no significant difference in calories received. Etiology-specific outcome data were generally not available for formal subgroup pooling. |
| Badpeyma et al. 2025 | Cause of ICU admission: poisoning 9 vs 4; respiratory 7 vs 6; cardiac 1 vs 3; neurologic 3 vs 6; sepsis 3 vs 5; other 5 vs 4. | Estimated energy target 25 kcal/kg/day; blenderized tube feeding plus whey protein supplementation to reach high-protein target. | Estimated energy target 25 kcal/kg/day; blenderized tube feeding with conventional-protein target. | Energy-comparable high-protein strategy. Etiology-specific outcome data were generally not available for formal subgroup pooling. |
| Bels et al. 2024 | Type of admission: medical 341 vs 308; surgical 124 vs 162; emergency surgery 107/124 vs 138/162; complications of elective surgery 17/124 vs 24/162. Diagnosis system: respiratory 173 vs 149; cardiovascular 128 vs 129; neurological 67 vs 82; trauma 42 vs 48; sepsis 21 vs 26; gastrointestinal 19 vs 22; other categories less frequent. | Isocaloric enteral formula, 1.3 kcal/mL and 0.10 g protein/mL; delivered energy similar to standard group. | Isocaloric enteral formula, 1.3 kcal/mL and 0.06 g protein/mL; delivered energy similar to high-protein group. | Isocaloric high-protein formula strategy. Etiology-specific outcome data were generally not available for formal subgroup pooling. |
| Chapple et al. 2021 | The study collected ICU admission diagnosis and severe head injury status, but detailed medical/surgical/trauma counts were not separately extractable from the main report available for this table. | High-protein EN formula containing 100 g protein/L; energy target delivered according to site/usual nutrition practice. | Standard-protein EN formula containing 63 g protein/L; energy target delivered according to site/usual nutrition practice. | Energy-comparable high-protein formula strategy; detailed achieved energy incompletely reported. Etiology-specific outcome data were generally not available for formal subgroup pooling. |
| Liu et al. 2024 | All enrolled patients met diagnostic criteria for severe pneumonia. | Energy supply set at 25 kcal/kg/day based on ideal body weight; target protein 1.8 g/kg/day. | Energy supply set at 25 kcal/kg/day based on ideal body weight; target protein 1.2 g/kg/day. | Isocaloric high-protein strategy. Etiology-specific outcome data were generally not available for formal subgroup pooling. |
| Hepduman et al. 2025 | Admission reasons: pneumonia 31/42; pulmonary edema 3/42; massive pulmonary embolism 2/42; diabetic foot ulcer 2/42; acute heart failure 2/42; pneumococcal meningitis 1/42; acute intra-alveolar hemorrhage 1/42. | Enteral nutrition using high-protein content to reach target calories; target calories otherwise comparable but not fully reported. | Enteral nutrition using lower-protein content to reach target calories; target calories otherwise comparable but not fully reported. | Energy-comparable high-protein strategy; achieved energy/protein incompletely reported. Etiology-specific outcome data were generally not available for formal subgroup pooling. |
| Doig et al. 2015 | Source of admission to ICU: operating room 76 vs 80; emergency department 66 vs 78; hospital ward 49 vs 36; other hospital 38 vs 38; transfer from ICU 5 vs 3; ICU readmission 1 vs 4. Surgical admission: emergency surgery 52 vs 51; elective surgery 27 vs 29. APACHE III admission diagnosis: respiratory 63 vs 60; gastrointestinal 49 vs 41; cardiovascular/vascular 48 vs 52; sepsis 25 vs 35; neurological 18 vs 29; trauma 17 vs 15; metabolic 5 vs 1; hematological 4 vs 2; orthopedic surgery 1 vs 1. | Standard nutrition plus IV amino-acid infusion adjusted to approximately 2.0 g/kg/day total protein; non-protein calories not deliberately changed. | Standard nutrition care without IV amino-acid supplementation. | Protein/amino-acid supplementation added to standard care; total energy slightly increased by amino acids. Etiology-specific outcome data were generally not available for formal subgroup pooling. |
| Dresen et al. 2021 | Diagnosis at ICU admission: general surgery 7 total (2 vs 5); abdominal surgery 10 total (5 vs 5); urogenital diseases 3 total (3 vs 0); ARDS 11 total (3 vs 8); trauma surgery 4 total (3 vs 1); cardiovascular surgery 7 total (5 vs 2). | Individualized nutrition targeting 100% measured resting energy expenditure by indirect calorimetry; target protein 1.8 g/kg/day. | Individualized nutrition targeting 100% measured resting energy expenditure by indirect calorimetry; target protein 1.2 g/kg/day. | Isocaloric high-protein strategy. Etiology-specific outcome data were generally not available for formal subgroup pooling. |
| Uyar et al. 2023 | Reason for ICU admission: respiratory 13 vs 14; neurological conditions 3 vs 6; AKI 2 vs 3; encephalitis 2 vs 0; cardiac arrest 1 vs 1; sepsis 2 vs 0; trauma 1 vs 1. | High-protein nutrition targeting 2.0 g/kg/day; caloric target/protocol not clearly extractable. | Standard-protein nutrition targeting 1.2 g/kg/day; caloric target/protocol not clearly extractable. | Likely energy-comparable high-protein strategy; energy details incompletely reported. Etiology-specific outcome data were generally not available for formal subgroup pooling. |
| Fetterplace et al. 2018 | Admission diagnosis: neurological conditions 13 vs 7; multi-trauma/head-trauma 7 vs 11; respiratory 1 vs 5; cardiac arrest 3 vs 1; sepsis 3 vs 1; cardiac surgery 0 vs 2; other 3 vs 3. | Volume-based EN protocol using high-protein formula plus protein supplement; target 25 kcal/kg/day; delivered energy 21 kcal/kg/day from nutrition and 23 kcal/kg/day total. | Standard hourly-rate EN protocol with 1.0 kcal/mL formula; target 25 kcal/kg/day; delivered energy 18 kcal/kg/day from nutrition and 21 kcal/kg/day total. | High-protein plus higher-energy co-intervention. Etiology-specific outcome data were generally not available for formal subgroup pooling. |
| Garcia-de-Lorenzo et al. 1997 | All included patients were septic ICU patients receiving parenteral nutrition; detailed medical/surgical/trauma counts were not reported in the extracted data. | Isocaloric TPN: 24 non-protein kcal/kg/day; 1.5 g amino acids/kg/day; 100:1 non-protein calories/g nitrogen. | Isocaloric TPN: 24 non-protein kcal/kg/day; 1.1 g amino acids/kg/day; 140:1 non-protein calories/g nitrogen. | Isocaloric parenteral amino-acid strategy. Etiology-specific outcome data were generally not available for formal subgroup pooling. |
| O’Keefe et al. 2025 | Patient type: trauma 430 overall (223 vs 207); necrotizing soft-tissue infection 39 overall (16 vs 23); other surgical conditions 31 overall (17 vs 14). | Early enteral protein supplementation aiming for higher protein intake, independent of enteral caloric intake; detailed energy delivery not fully reported. | Standard enteral nutritional support according to usual care; detailed energy delivery not fully reported. | Protein supplementation added to usual EN; energy exposure incompletely reported. Etiology-specific outcome data were generally not available for formal subgroup pooling. |
| Elkalawy et al. 2026 | All enrolled patients had traumatic brain injury and required mechanical ventilation. | High-protein enteral formula; energy requirements individualized, guided by indirect calorimetry; total calories reported to exceed 3000 kcal/day in discussion. | Standard-protein enteral formula; energy requirements individualized, guided by indirect calorimetry. | High-protein formula under IC-guided energy delivery; achieved energy details incompletely extractable. Etiology-specific outcome data were generally not available for formal subgroup pooling. |
| Heyland et al. 2023 | Primary ICU diagnosis: respiratory 275 vs 274; neurological 104 vs 93; sepsis 81 vs 90; trauma 59 vs 67; cardiovascular/vascular 56 vs 52; gastrointestinal 26 vs 37; other 23 vs 17; metabolic 13 vs 9; hematological 2 vs 5; burns 1 vs 5; orthopedic 3 vs 3; renal 1 vs 2; gynecological 1 vs 2. | High-dose protein prescription >=2.2 g/kg/day; energy delivery otherwise guided by standard ICU nutrition practice. | Usual-dose protein prescription <=1.2 g/kg/day; energy delivery guided by standard ICU nutrition practice. | Protein-dose strategy without a distinct hypocaloric protocol. Etiology-specific outcome data were generally not available for formal subgroup pooling. |
| Danielis et al. 2019 | Clinical diagnosis was collected, but detailed medical/surgical/trauma counts were not separately extractable from the available report. | Nutrition set to meet each patient's energy requirements; protein-fortified EN/PN composition; higher protein proportion. | Nutrition set to meet each patient's energy requirements; standard EN/PN composition. | Energy-comparable high-protein strategy; no energy difference except Day 1. Etiology-specific outcome data were generally not available for formal subgroup pooling. |
| Summers et al. 2025 | Admission diagnosis category: neurological 374 vs 350; respiratory 325 vs 360; cardiovascular 300 vs 310; trauma 204 vs 209; gastrointestinal 168 vs 190; other 162 vs 163; sepsis 148 vs 134. ICU source: emergency department 589 vs 618; operating theater 575 vs 595; hospital ward 227 vs 243; other hospital 181 vs 168; transfer from another ICU 109 vs 92. | Augmented-protein enteral formula strategy; achieved energy 17 kcal/kg IBW/day and 13 kcal/kg actual BW/day. | Usual-protein enteral formula strategy; achieved energy 17 kcal/kg IBW/day and 13 kcal/kg actual BW/day. | Isocaloric high-protein enteral strategy. Etiology-specific outcome data were generally not available for formal subgroup pooling. |
| Miri et al. 2025 | Admission diagnosis: trauma 6 vs 9; infection/sepsis 2 vs 2; neurologic 24 vs 16; cardiovascular 4 vs 0; surgical 1 vs 4; medical 6 vs 3; unspecified 7 vs 16. | High-protein enteral feeding target 1.5 g/kg/day; target energy described as comparable but detailed energy data not fully reported. | Standard enteral feeding target 1.0 g/kg/day; target energy described as comparable but detailed energy data not fully reported. | Energy-comparable high-protein strategy; achieved energy incompletely reported. Etiology-specific outcome data were generally not available for formal subgroup pooling. |
| Nakamura et al. 2021 | Basic diseases: sepsis 27 vs 30; heart failure 24 vs 21; stroke 29 vs 31; cardiopulmonary arrest 5 vs 7; post-surgery 21 vs 25; respiratory failure 21 vs 13; trauma 3 vs 7; endocrine/metabolic disorder 3 vs 1. | Target energy 20 kcal/kg/day; high-protein EN product providing 1.8 g protein per 20 kcal target. | Target energy 20 kcal/kg/day; medium-protein EN product providing 0.9 g protein per 20 kcal target. | Isocaloric high-protein strategy. Etiology-specific outcome data were generally not available for formal subgroup pooling. |
| Rugeles et al. 2013 | Reason for ICU admission: respiratory disease 16 vs 14; CNS disorder 13 vs 12; cardiac disease 2 vs 4; gastrointestinal disease 0 vs 3; other 9 vs 7. | Prescribed hyperproteic hypocaloric EN: 15 kcal/kg/day with 1.7 g/kg/day protein; achieved approx. 12 kcal/kg/day. | Prescribed isocaloric EN: 25 kcal/kg/day with 20% calories as protein; achieved approx. 14 kcal/kg/day. | Hypocaloric-hyperproteic strategy. Etiology-specific outcome data were generally not available for formal subgroup pooling. |
| Jakob et al. 2017 | Eligible population included medical and surgical ICU patients expected to require prolonged ICU stay and tube feeding; detailed etiology counts were not separately extractable from the available table. | Peptamen AF, 1.5 kcal/mL, 25% energy from protein; caloric goal adjusted by indirect calorimetry; achieved energy 18.0 kcal/kg/day. | Isosource Energy, 1.5 kcal/mL, 16% energy from protein; caloric goal adjusted by indirect calorimetry; achieved energy 19.7 kcal/kg/day. | Isocaloric high-protein formula strategy. Etiology-specific outcome data were generally not available for formal subgroup pooling. |
| van Zanten et al. 2018 | Admission diagnosis: medical 9 vs 8; surgical 10 vs 11; surgical nontrauma 4 vs 4; surgical trauma 6 vs 7; trauma 9 vs 10; trauma nonsurgical 3 vs 3. Surgical trauma patients were included in both surgical and trauma subgroups in the source report. | Very-high intact-protein formula, 1.25 kcal/mL and 8 g protein/100 kcal; no increase in energy intake. | Standard high-protein formula, 1.25 kcal/mL and 5 g protein/100 kcal. | Isocaloric high protein-to-energy formula strategy. Etiology-specific outcome data were generally not available for formal subgroup pooling. |
| Parasuraman et al. 2025 | Most admission diagnoses were pneumonia (43.3%). Detailed group-level etiology counts were not separately extractable from the available report. | Normocaloric EN: about 25 kcal/kg/day; high-protein target 1.5 g/kg/day. | Normocaloric EN: about 25 kcal/kg/day; standard-protein target 1.0 g/kg/day. | Isocaloric/normocaloric high-protein strategy. Etiology-specific outcome data were generally not available for formal subgroup pooling. |
| Wu et al. 2025 | Patients were enrolled from a surgical ICU. Diagnosis at ICU admission was reportedly comparable between groups, but detailed category counts were provided in supplementary material and were not available in the extracted main text. | Target calories 25 kcal/kg/day for 7 days; target protein 2.5-3.0 g/kg/day, at least 1.5 g/kg/day if not tolerated; EN preferred with PN protein supplement if needed. | Target calories 25 kcal/kg/day for 7 days; target protein 1.2-1.5 g/kg/day. | Isocaloric high-protein strategy. Etiology-specific outcome data were generally not available for formal subgroup pooling. |
| Zhang et al. 2022 | Admission category: medical 9 vs 8; surgical 12 vs 12. Severe sepsis: 16 vs 13. | Intensive nutrition treatment; actual energy 33.46 +/- 2.78 kcal/kg/day and protein 1.70 +/- 0.21 g/kg/day. | Standard nutrition treatment; actual energy 25.75 +/- 4.81 kcal/kg/day and protein 1.06 +/- 0.21 g/kg/day. | High-protein plus higher-energy co-intervention. Etiology-specific outcome data were generally not available for formal subgroup pooling. |
| Zhou et al. 2006 | All enrolled patients had severe stroke. | High-protein EN formula; equal non-protein calories 104.5 kJ/kg/day. | Standard EN formula; equal non-protein calories 104.5 kJ/kg/day. | Isocaloric high-protein formula strategy. Etiology-specific outcome data were generally not available for formal subgroup pooling. |

**Supplementary Table S3. Detailed ICU admission etiology, case mix, and energy-delivery strategies of randomized controlled trials included in the quantitative synthesis.** This table provides additional details on ICU admission etiology or case mix, including medical, surgical, trauma, neurological, respiratory, septic, or mixed ICU populations when reported by the primary studies. When etiology-specific numbers or proportions were available, they were extracted directly; otherwise, the study population was classified according to the stated inclusion criteria or dominant enrolled population. Energy-delivery information includes prescribed or target energy intake, achieved energy intake when available, and whether the higher-protein intervention was delivered under an energy-comparable, hypocaloric - hyperproteic, higher-energy co-intervention, or unclear/add-on feeding paradigm. Achieved energy intake was prioritized over prescribed targets when both were reported. If detailed achieved energy intake was not reported, this was indicated as incompletely reported.
Abbreviations: EN, enteral nutrition; PN, parenteral nutrition; TPN, total parenteral nutrition; IV, intravenous; ICU, intensive care unit; IC, indirect calorimetry; IBW, ideal body weight; ABW, actual body weight; TBI, traumatic brain injury; GI, gastrointestinal; VAP, ventilator-associated pneumonia; ICU-AW, intensive care unit-acquired weakness.

| **Excluded study** | **No. of studies retained** | **Pooled MD, days** | **95% CI** | **I²** | **Interpretation** |
| --- | --- | --- | --- | --- | --- |
| None | 5 | -0.92 | -2.37 to 0.52 | 87% | Substantial heterogeneity remained |
| Azevedo et al. 2019 | 4 | -1.09 | -2.74 to 0.56 | 90% | Heterogeneity remained substantial |
| Badpeyma et al. 2025 | 4 | -1.04 | -2.87 to 0.78 | 90% | Heterogeneity remained substantial |
| Elkalawy et al. 2026 | 4 | -0.21 | -0.99 to 0.58 | 41% | Heterogeneity was substantially reduced |
| Summers et al. 2025 | 4 | -1.35 | -2.75 to 0.05 | 70% | Heterogeneity was reduced but remained moderate/substantial |
| Nakamura et al. 2021 | 4 | -0.82 | -2.58 to 0.94 | 89% | Heterogeneity remained substantial |

**Supplementary Table S4. Leave-one-out influence analysis for duration of mechanical ventilation among energy-comparable or target-comparable trials.** MDs were calculated as the higher-protein group minus the lower/standard-protein group; therefore, negative values indicate shorter mechanical ventilation duration in the higher-protein group. A random-effects inverse-variance model was used. CI, confidence interval; MD, mean difference.

| **Study** | **Outcome domain** | **Anatomical site** | **Muscle-related measure** | **Time point / follow-up** | **Descriptive finding** | **Synthesis decision** | **Reason / notes** |
| --- | --- | --- | --- | --- | --- | --- | --- |
| Azevedo et al. 2019 | Muscle function | Handgrip strength | Handgrip strength / physical function-related outcome | Post-ICU / follow-up period | No quantitative pooling; reported as functional recovery-related outcome | Descriptive only | Functional outcome; not directly comparable with ultrasound-based muscle mass measures. |
| Liu et al. 2024 | Peripheral muscle; diaphragm | Quadriceps; diaphragm | Quadriceps atrophy/thickness and diaphragm ultrasound indices | Day 5 / Day 10 | Reported selected ultrasound-based quadriceps and diaphragm indices; quantitative pooling was not feasible | Descriptive only | Eligible by protein-difference criterion, but measurement site and construct differed from other studies. |
| Hepduman et al. 2025 | Diaphragm; peripheral muscle | Diaphragm; mid-arm muscle circumference | Diaphragm thickness and mid-arm muscle circumference | During mechanical ventilation follow-up | Reported muscle/diaphragm changes, but comparable pooled data were insufficient | Descriptive only | Different anatomical site and measurement structure from other studies. |
| Dresen et al. 2021 | Peripheral muscle | Quadriceps | Quadriceps muscle layer thickness | Late ICU phase / follow-up to 4 weeks | Reported quadriceps muscle loss/preservation outcome | Descriptive only | Different illness phase and measurement schedule; not pooled. |
| Uyar et al. 2023 | Diaphragm; peripheral muscle | Diaphragm; rectus femoris | Diaphragm thickness/function and rectus femoris thickness | Up to 15 days | Reported diaphragm and rectus femoris changes | Descriptive only | Different anatomical sites and combined respiratory/peripheral muscle constructs. |
| Fetterplace et al. 2018 | Peripheral muscle | Quadriceps | Quadriceps muscle layer thickness | ICU feeding period | Reported muscle-related outcome but not sufficiently comparable for final pooling | Descriptive only | Eligible study, but quantitative synthesis not performed under final strict criteria. |
| Elkalawy et al. 2026 | Peripheral muscle | Rectus femoris | Rectus femoris cross-sectional area and pennation angle | ICU follow-up | Reported selected rectus femoris ultrasound indices; quantitative pooling was not feasible | Descriptive only | Specialized TBI population; different muscle site and indices. |
| Miri et al. 2025 | Peripheral muscle | Biceps / upper arm | Biceps muscle thickness and mid-upper arm circumference | Up to 21 days | Reported upper-limb muscle-related outcomes | Descriptive only | Different anatomical site from quadriceps/rectus femoris studies. |
| Nakamura et al. 2021 | Peripheral muscle | Femoral muscle | Femoral muscle volume | By Day 10 / follow-up | Reported femoral muscle volume outcome | Descriptive only | Muscle volume measure differs from ultrasound thickness/atrophy outcomes. |
| Parasuraman et al. 2025 | Peripheral muscle | Multiple peripheral muscle sites | Muscle thickness, cross-sectional area, echogenicity, and pennation angle | First 7 ICU days | Reported multiple ultrasound-based muscle morphology indices | Descriptive only | Small sample; multiple indices and sites; not pooled. |
| Wu et al. 2025 | Peripheral muscle; functional weakness | Skeletal muscle / ICU-acquired weakness | Muscle wasting and ICU-acquired weakness | ICU nutrition period | Reported muscle wasting and ICU-AW-related outcomes | Descriptive only | Both groups were high-protein; outcome definitions differed from other studies. |
| Zhang et al. 2022 | Diaphragm | Diaphragm | Diaphragm atrophy and respiratory mechanics | Prolonged mechanical ventilation follow-up | Reported diaphragm atrophy/respiratory mechanics outcomes | Descriptive only | Diaphragm morphology and respiratory mechanics were not pooled with peripheral muscle outcomes. |

**Supplementary Table S5. Descriptive summary of muscle-related outcomes.** This table summarizes muscle mass, diaphragm-related, and muscle function outcomes reported across eligible studies. Quantitative synthesis was not performed because the outcomes were not sufficiently comparable in terms of anatomical site, measurement technique, or assessment time point. Ferrie 2016 was excluded from the final descriptive table because it did not meet the prespecified ≥0.3 g/kg/day protein-separation criterion.

**Supplementary Figure S1. Sensitivity and exploratory subgroup analyses.** A, 28/30-day all-cause mortality after excluding the study with clearly non-comparable energy delivery; B, 60-day all-cause mortality after excluding the study with clearly non-comparable energy delivery; C, 90-day all-cause mortality after excluding the add-on amino acid trial with unclear total energy exposure; D, exploratory subgroup analysis of duration of mechanical ventilation according to energy-delivery strategy; E, exploratory subgroup analysis of ICU length of stay according to energy-delivery strategy. For dichotomous outcomes, risk ratios were pooled using random-effects Mantel–Haenszel models. For continuous outcomes, MDs were calculated as higher-protein minus lower/standard-protein provision; therefore, negative values indicate shorter duration or length of stay in the higher-protein group. Random-effects inverse-variance models were used for continuous outcomes.
Abbreviations: CI, confidence interval; MD, mean difference.

**Supplementary Figure S2. Additional nutrition/metabolic and safety outcomes.** A, Day 5 nitrogen balance; B, gastrointestinal intolerance composite outcome. For Day 5 nitrogen balance, MDs were calculated as higher-protein minus lower/standard-protein provision; therefore, positive values indicate higher nitrogen balance in the higher-protein group. For gastrointestinal intolerance, risk ratios compare higher-protein with lower/standard-protein provision; values greater than 1 indicate a higher risk in the higher-protein group. Random-effects models were used. Abbreviations: CI, confidence interval; MD, mean difference.
